# Cultural and Environmental Predictors of Pre-European Deforestation on Pacific Islands

**DOI:** 10.1371/journal.pone.0156340

**Published:** 2016-05-27

**Authors:** Quentin D. Atkinson, Ties Coomber, Sam Passmore, Simon J. Greenhill, Geoff Kushnick

**Affiliations:** 1 School of Psychology, University of Auckland, Private Bag 92019, Auckland, 1142, New Zealand; 2 Max Planck Institute for History and the Sciences, Kahlaische Strasse 10, 07745, Jena, Germany; 3 School of Culture, History and Language, ANU College of Asia and the Pacific, The Australian National University, Canberra, Australian Capital Territory, Australia; 4 ARC Center of Excellence for the Dynamics of Language, The Australian National University, Canberra, Australian Capital Territory, Australia; 5 School of Archaeology and Anthropology, ANU College of Arts and Social Sciences, The Australian National University, Canberra, Australian Capital Territory, Australia; Universitat Pompeu Fabra, SPAIN

## Abstract

The varied islands of the Pacific provide an ideal natural experiment for studying the factors shaping human impact on the environment. Previous research into pre-European deforestation across the Pacific indicated a major effect of environment but did not account for cultural variation or control for dependencies in the data due to shared cultural ancestry and geographic proximity. The relative importance of environment and culture on Pacific deforestation and forest replacement and the extent to which environmental impact is constrained by cultural ancestry therefore remain unexplored. Here we use comparative phylogenetic methods to model the effect of nine ecological and two cultural variables on pre-European Pacific forest outcomes at 80 locations across 67 islands. We show that some but not all ecological features remain important predictors of forest outcomes after accounting for cultural covariates and non-independence in the data. Controlling for ecology, cultural variation in agricultural intensification predicts deforestation and forest replacement, and there is some evidence that land tenure norms predict forest replacement. These findings indicate that, alongside ecology, cultural factors also predict pre-European Pacific forest outcomes. Although forest outcomes covary with cultural ancestry, this effect disappears after controlling for geographic proximity and ecology. This suggests that forest outcomes were not tightly constrained by colonists’ cultural ancestry, but instead reflect a combination of ecological constraints and the short-term responses of each culture in the face of those constraints.

## Introduction

The role of culture and ecology in shaping the environmental impact of Pacific peoples has captured the interest of scholars from the earliest ethnographies [1] to more recent debates around environmental determinism [2–7]. Following their remarkable expansion across the Pacific [8], Austronesian-speaking cultures with different social institutions, norms and means of production were faced with the challenge of survival on islands varying in size, isolation, climate and ecology. Whilst some groups were able to sustainably manage resources and flourish [6], others experienced environmental degradation and possibly even societal collapse [9,10], *cf*.[11]. Each island can thus be seen as a natural experiment in human-environment interactions [12].

The most conspicuous impact of Pacific peoples on their environments was land clearance and tree felling for timber, fuel and agriculture. Early European explorers observed varying degrees of deforestation, with native forests intact on some islands, whilst other islands were completely deforested or reforested with introduced species [6]. Previous work has tested the link between environmental variables and forest outcomes as recorded by European explorers [3]. Deforestation and/or forest replacement was found to decrease with island rainfall, elevation, area, volcanic ash fallout, Asian dust transport and makatea terrain, and increase with island latitude, age and isolation. However, this analysis did not account for the potential effects of culture [6,12,13], nor did it control for potential statistical dependencies in forest outcomes due to diffusion or inheritance of culturally transmitted beliefs and practices–a statistical trap known as “Galton’s problem” [14].

Here, we address these gaps in two ways. First, we seek to overcome Galton’s problem by quantifying and controlling for the impact of cultural ancestry and diffusion on pre-European Pacific forest outcomes. To the extent that culture is important, forest outcomes may not represent independent datapoints because Pacific island societies share a common cultural ancestry derived from the Austronesian expansion. Additionally, more recent cultural diffusion or regional differences in island ecology can create spatial dependencies in the data. Polynesian plant introductions, for example, are known to be geographically patterned [15]. By combining forest outcome data with information on the location and linguistic affiliation of each island’s inhabitants, we can map forest outcomes onto the Austronesian language tree [8] and use comparative phylogenetic methods [16] to quantify and control for non-independence due to shared ancestry and geographic proximity (Materials and Methods). We use this approach to: a) test whether putative relationships between ecology and forest outcomes are robust to controls for any non-independence in the data; and b) quantify the extent to which forest outcomes in the Pacific reflect geographic location and/or cultural ancestry as tracked by language phylogeny. Stong phylogenetic signal or ‘phylogenetic inertia’ [17] in the forest outcome data is consistent with cultural ancestry constraining society’s impact on their environment, whilst a lack of phylogenetic signal suggests societies tend to quickly adapt to local conditions.

Second, we use the rich ethnographic record of Pacific societies to test the effects of two cultural variables thought to influence land use.

### Agricultural intensification

Populations across the Pacific vary as to whether and how they intensified agricultural production. Following Kirch [2,18], we categorize the mode of agricultural intensification from societies at each site as follows: wet (e.g., irrigation), arboricultural (tree crops), dry (e.g., rock gardens and mulching to shorten fallow periods) or none (S1 and S2 Tables). *Wet intensification* has been argued to reduce pressure on land clearance and hence reduce deforestation by providing increased yields, transforming otherwise marginal agricultural land such as swamps, and minimizing fallow periods [6]. However, wet intensification may also act to increase deforestation by fuelling population growth and promoting permanent land clearance, preventing regeneration. The same predictions can be made for *dry intensification* although any effect is likely to be weaker because yield gains are generally lower than for wet intensification. *Arboricultural intensification* should be associated with reduced deforestation (as there is less need for cleared land) and increased forest replacement from introduced species [6].

### Land tenure norms

Norms of land ownership show considerable variation across Pacific populations and tend to be inherited vertically down cultural lineages [19]. For societies from each island location in our dataset, we coded land tenure norms based on the presence or absence of *individual* or *elite* ownership versus *other* (corporate or no) ownership (S3 and S4 Tables) and evaluate competing hypotheses relating to their effects on forest outcomes. One hypothesis is that individual or elite ownership circumvents the ‘tragedy of the commons’, resulting in less deforestation and more replacement than other forms of ownership [20,21]. Another hypothesis holds that because elites are more easily able to meet their short-term needs, they are better placed to invest in and enforce long-term goals, leading to less deforestation and more replacement in societies with elite ownership in particular. Conversely, elites may have little knowledge of local conditions and less incentive to manage resources than individuals or groups because their livelihoods do not depend on them [22]. Under this hypothesis, elite ownership should be associated with more deforestation and less replacement compared to individual or other forms of ownership.

## Results and Discussion

### Cultural ancestry and forest outcomes

**Fig 1:**
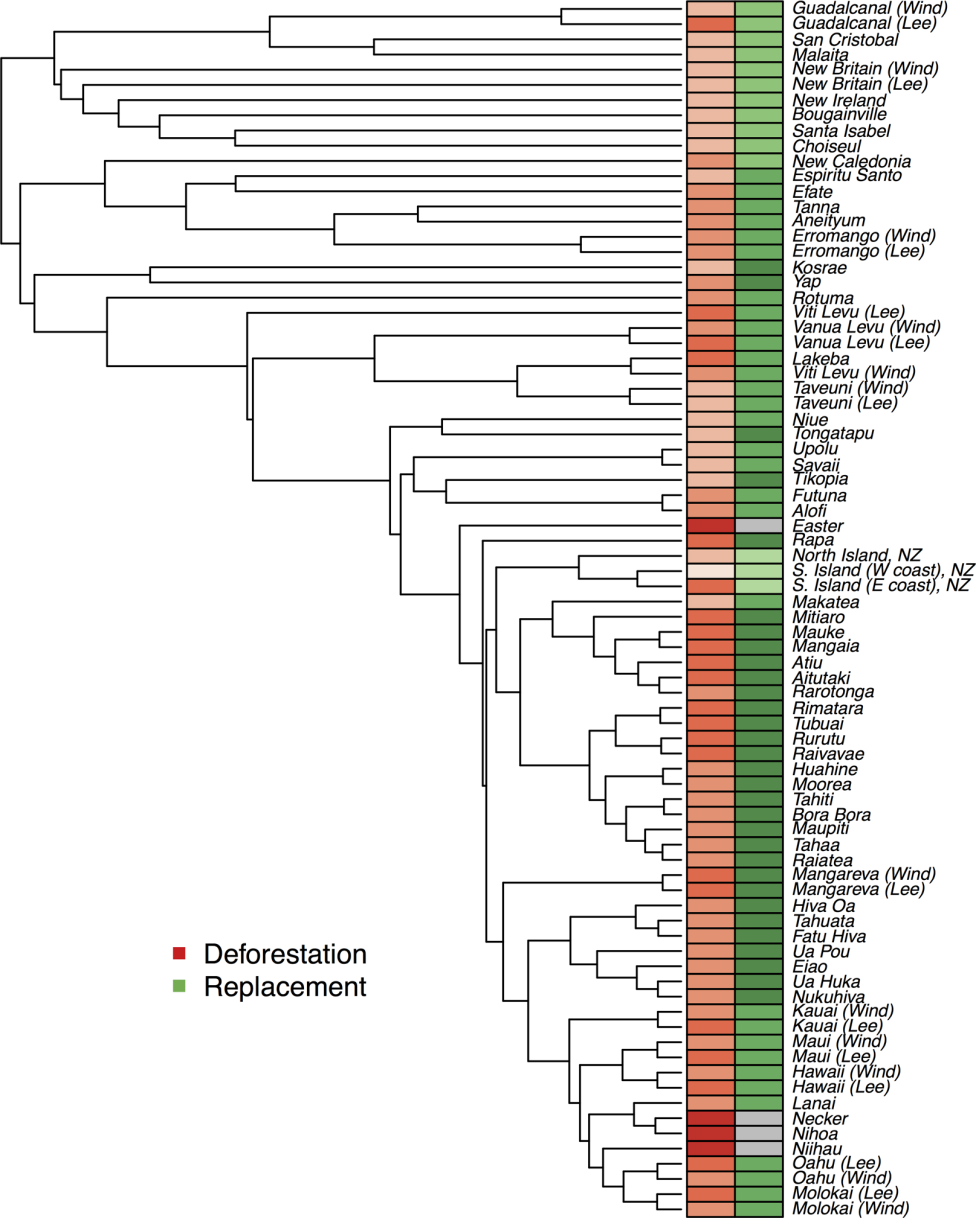
Pre-European Pacific forest outcomes across the Austronesian language family. Deforestation (red) and replacement (green) scores mapped onto the Austronesian Maximum Clade Crediblity tree for languages sampled from across 80 sites. Darker shading equates to increased deforestation/replacement.

Fig 1 shows deforestation and forest replacement data mapped onto the Austronesian language family tree (see Materials and Methods). Forest outcome data is based on deforestation and modified forest replacement scores from ref. [3] (Materials and Methods). We quantified the degree of phylogenetic signal (non-independence due to shared cultural ancestry) in the forest outcome data using Pagel’s lambda (λ)[23]. Both deforestation (λ_deforestation_ = 0.543, n = 80, p<0.001) and forest replacement (λ_replacement_ = 1.00, n = 76, p<0.001) show clear phylogenetic signal. That is, islands inhabited by more closely related cultures have more similar forest outcomes.

**Fig 2:**
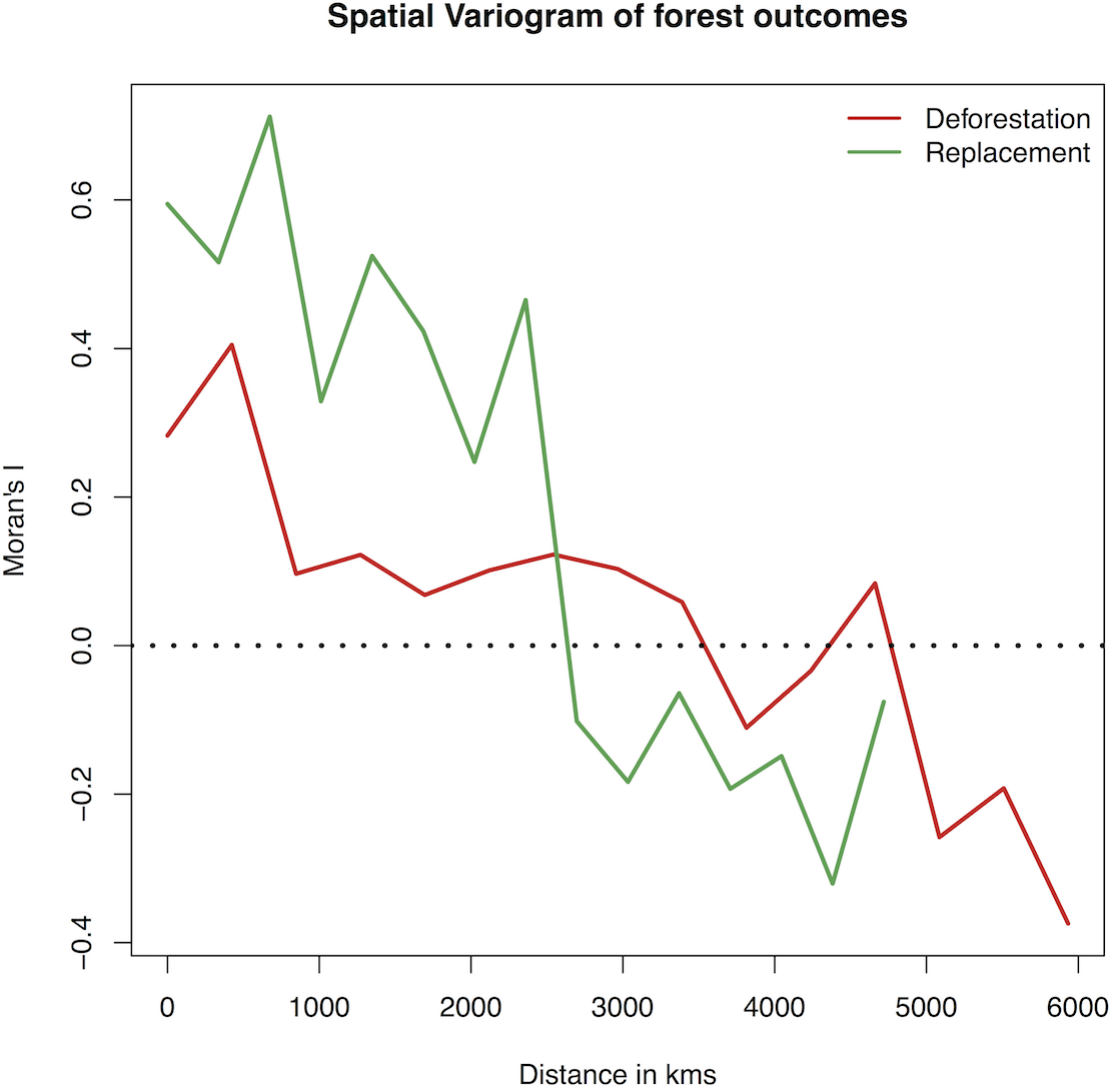
Spatial autocorrelation in Pre-European Pacific forest outcomes. Spatial variogram showing degree of spatial auto-correlation in deforestation (green) and replacement (red) as a function of geographic proximity (distance between islands).

One explanation for this pattern is phylogenetic inertia due to the vertical inheritance of putative cultural drivers of Pacific forest outcomes, such as land ownership, agriculture or particular crops or animals. However, mapping the forest outcome data reveals that islands that are geographically closer to one another have more similar deforestation and replacement scores (Fig 2 and S1 Fig). Since closely related languages are more likely to be geographic neighbours, phylogenetic signal could also arise due to cultural diffusion between neighbours or regional patterning of ecological predictors, without the inheritance of traits linked to forest outcomes. In order to tease apart these different processes, we use a phylogenetic generalized least squares spatial (PGLS-spatial) [16] approach to combine ecological predictors, cultural ancestry and geographic proximity into a single analysis.

### Ecological predictors of forest outcomes

Table 1 presents results from our PGLS-spatial analysis of forest outcomes (Materials and Methods), simultaneously estimating and controlling for the effects of cultural ancestry (*λ*′), geographic proximity (*ϕ*) and the putative ecological predictors previously linked to forest outcomes [3]. We use model averaging [24] to calculate relative variable importance (RVI) across all possible model combinations. In order to control for uncertainty in the reconstructed language phylogeny, analyses are averaged across 100 language trees from a Bayesian posterior distribution of trees (see Materials and Methods).

**Table 1:** Ecological predictors of forest outcomes.

Deforestation				Forest Replacement			
**Predictor**	**RVI**	**Beta**	**95% C.I.**	**Predictor**	**RVI**	**Beta**	**95% C.I.**
**Log(Rainfall)**	1	-1.599	-2.086, -1.111	**Log(Area)**	0.998	-0.124	-0.184, -0.063
Log(Elevation)	0.911	-0.689	-1.083, -0.294	**Tephra = 3**	0.987	-1.215	-2.221, -0.208
**Log(Isolation)**	0.895	0.263	0.076, 0.45	**Log(Isolation)**	0.6	0.057	0.001, 0.113
**Abs. Latitude**	0.642	0.019	0.002, 0.037	**Abs. Latitude**	0.547	-0.028	-0.056, 0.001
**Tephra = 2**	0.626	-0.624	-1.218, -0.03	**Tephra = 2**	0.543	-0.736	-1.543, 0.072
**Log(Area)**	0.373	-0.160	-0.375, 0.056	**Age**	0.257	-0.038	-0.106, 0.029
Tephra = 3	0.337	-0.226	-0.624, 0.172	**Dust**	0.207	-0.001	-0.002, 0.001
% Makatea	0.235	-0.259	-1.68, 1.162	**Log(Elevation)**	0.157	0.019	-0.124, 0.163
Dust	0.198	-0.0003	-0.001, 0.001	Log(Rainfall)	0.152	0.008	-0.062, 0.077
Age	0.163	0.059	-0.223, 0.342	**% Makatea**	0.15	0.022	-0.352, 0.395
**Dependency**		**Mean**	**p-value**	**Dependency**		**Mean**	**p-value**
Cultural (λ')		0.017	0.957	Cultural (λ')		0	1.0
Geographic (ϕ)		0.082	0.932	Geographic (ϕ)		1.0	<0.001
Independent (γ)		0.901	-	Independent (γ)		0	-

Table shows relative variable importance, Akaike weighted beta estimate and 95% confidence interval for PGLS analysis (Materials and Methods) of the effects of putative ecological predictors, phylogeny and geographic proximity on deforestation (n = 76) and forest replacement (n = 72). Previously identified significant predictors of deforestation and forest replacement are shown in bold. All values integrate over phylogenetic and sampling uncertainty across 100 replicates from our posterior distribution of language trees.

Table 1 shows that, after controlling for geographic proximity and ecology, cultural ancestry no longer predicts deforestation or forest replacement. We also find no consistent effect of geographic proximity on deforestation. This suggests that covariation of cultural ancestry with deforestation is largely accounted for by the effects of ecological predictors, which themselves covary with cultural ancestry (S5 Table). However, we find a clear and consistent effect of geographic proximity on forest replacement. That is, islands that are closer to one another have more similar forest replacement scores. Follow-up analyses reveal that cultural ancestry remains a significant predictor of forest replacement in models with only cultural ancestry and ecological predictors and no geographic effects (S6 Table), but is not a significant predictor of forest replacement in models with only cultural ancestry and geographic effects and no ecological predictors (S7 Table). This implies that covariation of forest replacement with cultural ancestry is due largely to spatial patterning in the forest replacement data that is unlinked to our ecological predictors.

The model averaging results summarized in Table 1 indicate five predictors of deforestation have a confidence interval that excludes zero and these all have an RVI greater than 60%–rainfall, elevation, isolation, absolute latitude and tephra level 2 (see S8 Table for variable definitions). A model including these five predictors explains approximately 69% of the variance in deforestation (R^2^ = 0.689). For forest replacement, three predictors have a confidence interval that excludes zero–island area, tephra level 3 and isolation. A further two predictors have an RVI greater than 50% but their confidence interval includes zero at the margins–latitude and tephra level 2. A model including these top five predictors explains approximately 40% of the variance in forest replacement (R^2^ = 0.401).

These findings highlight revealing similarities and differences with previous work [3]. Confirming previous research, we find a strong negative relationship between rainfall and deforestation scores. This is consistent with the proposal that higher rainfall increases plant growth rates and forest regeneration. Growth rates are also expected to be influenced by absolute latitude (as a proxy for temperature) [3], and our findings confirm a positive association between absolute latitude and deforestation. We also find some support for the finding that higher latitudes see less forest replacement, although the 95% confidence interval includes zero. Such an association has been linked to the unviability of the main tree crops (breadfruit and Tahitian chestnut) at higher latitudes [3].

Three of the putative ecological predictors are proposed to impact forest outcomes by affecting soil nutrient levels [3]. Since soil nutrients are lost with time, island age is expected to predict increased deforestation. Conversely, volcanic dust fallout (tephra) and continental dust fallout (dust) act to replenish nutrients and should reduce deforestation. We find support for less deforestation with moderate tephra levels (level 2) but not higher tephra levels (level 3), whilst less forest replacement is associated with high and, less clearly, moderate tephra levels. We do not find support for the previously identified relationships between forest replacement and both island age and continental dust fallout.

A combination of various mechanisms are proposed to underlie the effect of the remaining four ecological variables. Consistent with previous findings, isolation (distance from other islands) is associated with increased deforestation and forest replacement. This may reflect the potential for trade and emigration to temper resource depletion on less isolated islands [3]. Percentage of makatea (uplifted reef that holds little soil and is difficult to walk on) was previously found to predict decreased forest replacement, purportedly because it makes tree growth and access difficult in these areas [3]. However, our analyses find no support for a relationship between makatea coverage and deforestation or forest replacement after controlling for other ecological predictors. Island area is hypothesized to predict decreased deforestation and forest replacement because of a combination of greater species diversity, the buffering effect of a larger land mass and greater area/perimeter ratio (greater population densities on the coast have more forested area at their disposal) [3]. Our findings confirm the relationship linking greater island area with lower forest replacement, but do not support the previous finding that greater island area predicts reduced deforestation. Finally, increased elevation was previously found to predict decreased forest replacement and, when area is removed as a predictor, decreased deforestation. This was suggested to be due to several factors–increased orographic rain makes coastal areas effectively wetter than is indicated by their rainfall and also captures atmospheric dust, erosion from high elevations replenishes soil nutrients in lowland areas, and agriculture is less likely at higher elevations due to climate and access [3]. Our results confirm an association between increased elevation and decreased deforestation but we do not find support for an association between elevation and forest replacement.

### Cultural predictors of forest outcomes

To test whether mode of agricultural intensity and land ownership predict forest outcomes, we repeated the above model-selection procedure, combining the top five ecological predictors of deforestation and forest replacement with five cultural variables (see Materials and Methods)–three modes of agricultural intensification (wet, dry and arboriculture, with no intensification as the baseline) and two land ownership systems (individual and elite, with ‘other’ as the baseline). Table 2 shows our findings presented in the same format as Table 1.

**Table 2:** Ecological and cultural predictors of forest outcomes.

Deforestation				Forest Replacement			
**Predictor**	**RVI**	**Beta**	**95% C.I.**	**Predictor**	**RVI**	**Beta**	**95% C.I.**
Log(Rainfall)	1.000	-0.727	-0.936, -0.518	*Arboriculture*	0.999	0.819	0.737, 1.00
*Wet intens*.	0.826	0.470	0.197, 0.744	Log(Area)	0.633	-0.029	-0.048, -0.023
Log(Elevation)	0.762	-0.167	-0.278, -0.055	Tephra = 2	0.451	-0.719	-1.028, -0.041
Tephra = 2	0.580	-0.584	-1.173, 0.004	Tephra = 3	0.406	-0.819	-1.111, -0.015
Log(Isolation)	0.566	0.090	0.003, 0.177	*Elite Ownership*	0.288	-0.254	-0.325, 0.648
Abs. Latitude	0.257	0.014	-0.015, 0.043	*Ind*. *Ownership*	0.251	-0.061	-0.080, 0.055
*Ind*. *Ownership*	0.242	-0.147	-0.485, 0.19	Log(Isolation)	0.218	0.009	-0.004, 0.018
*Dry intens*.	0.237	0.150	-0.158, 0.458	Abs. Latitude	0.211	-0.011	-0.037, 0.006
*Arboriculture*	0.209	-0.139	-0.585, 0.307	*Wet intens*.	0.192	0.012	-0.006, 0.097
*Elite Ownership*	0.195	0.051	-0.385, 0.486	*Dry intens*.	0.179	-0.004	-0.013, 0.110
**Dependency**		**Mean**	**p-value**	**Dependency**		**Mean**	**p-value**
Cultural (λ')		0.007	0.902	Cultural (λ')		0.00	1.0
Spatial (ϕ)		0.307	0.698	Spatial (ϕ)		1.0	<0.001
Independent (γ)		0.686	-	Independent (γ)		0.0	-

As for Table 1 but predicting deforestation (n = 80) and forest replacement (n = 76) from the five most important ecological predictors together with the agricultural intensification and land tenure cultural predictors. Cultural predictors in *italics*.

Table 2 shows that, controlling for ecology and potential dependencies in the data, wet intensification is the second most important predictor of deforestation behind rainfall, with a reliance on wet intensification predicting greater deforestation scores. This is contrary to the prediction that more efficient wet intensification methods, usually irrigated taro cultivation, will generally reduce pressure on forest resources [6]. It is possible that wet intensification has this effect in some circumstances [6], but the general pattern we observe is increased deforestation in the presence of wet intensification. This could occur if deforestation prevents regeneration of native forest by permitting the continual cultivation of cleared land. However the wetland environments required for irrigation are generally limited in area. An alternative explanation is that wet intensification is associated with increased population size, perhaps as a result of increased food production capacity, which drives further deforestation for land clearance and forest resources. We do not find evidence that any of the other cultural variables predict deforestation scores. A model combining the four predictors with a confidence interval that excludes zero—rainfall, wet intensification, elevation and isolation–plus tephra level 2 which has a confidence interval that includes zero at the margins but an RVI of 58%, explains approximately 71% of the variance in deforestation (R^2^ = 0.709).

Table 2 shows that a reliance on arboriculture predicts increased forest replacement, as expected if forest replacement is driven by reliance on introduced tree crops for food production. Indeed, arboriculture is the most important predictor of forest replacement score according to our analyses. A model combining the four predictors with a confidence interval that excludes zero—arboriculture, island area and tephra level 2 and tephra level 3 –explains 41% of the variance in deforestation (R^2^ = 0.410). Whilst our model averaging procedure did not identify any other important cultural predictors, bivariate analyses of the effect of each cultural predictor show that elite land ownership predicts forest replacement on its own (S9 Table). It is possible the association between elite land ownership and forest replacement is mediated by an increased reliance on arboriculture in these cultures. Consistent with this explanation, a PGLS bivariate regression shows that elite land ownership predicts the presence of arboriculture (95% C.I. = 0.701–1.053). Further, when the model averaging procedure is repeated with arboriculture excluded, elite ownership emerges as the most important predictor (RVI = 0.988, 95% C.I. = 0.271–0.941; S10 Table). This pattern of results is consistent with elite ownership increasing forest replacement via increased reliance on arboriculture, perhaps due to greater willingness to invest in replanting introduced species.

The inclusion of cultural predictors in our model did not appreciably change the proportion of variation in both deforestation and forest replacement that is attributable to cultural ancestry and geographic proximity. Deforestation continues to show no consistent effect of cultural ancestry or geographic proximity and forest replacement continues to show no effect of cultural ancestry but a strong effect of geographic proximity. This indicates that the geographic effect on forest replacement is not due to regional patterns of agricultural intensification or land ownership. Spatial effects in forest replacement could reflect the geographic diffusion of tree crops across the Pacific [15] or unmodelled regional ecological variation.

The inferred importance of ecological predictors and the direction of their effects on forest outcomes were also generally unaffected by the inclusion of cultural predictors. An interesting exception was that absolute latitude ceased to emerge as an important predictor of both deforestation and forest replacement. This suggests the relationship between absolute latitude and forest outcomes could be due in part to latitudinal variation in mode of agricultural intensification. Consistent with this explanation, wet intensification (linked to more deforestation) is less prevalent closer to the equator (Beta = 0.020; 95% C.I. = -0.008–0.047) and arboriculture (linked to more replacement) is more prevalent (Beta = -0.021; 95% C.I. = -0.053–0.011), but the 95% confidence intervals both include zero, indicating these associations are weak.

## Conclusion

Mapping the forest outcome data onto the Austronesian language tree shows that cultural ancestry alone is a strong predictor of both deforestation and forest replacement in the pre-European Pacific. Although this is consistent with cultural phylogenetic inertia [17] constraining forest outcomes, when spatial effects and ecological predictors are included in our models, the covariation with cultural ancestry disappears. This suggests that forest outcomes for each of the groups in our sample were not constrained by their cultural ancestry, but were instead the result of relatively rapid responses to the local social and ecological environment.

We also find a strong effect of geographic proximity on forest replacement (but not deforestation), which persists even after controlling for the candidate ecological and cultural predictors in our model. This effect may be due to unmodelled regional patterning of ecological factors, such as growing season or hurricane strength, or the geographic diffusion of cultural traits linked to forest replacement, such as knowledge of arboriculture or the exchange of plant varieties [15].

In addition to quantifying the relative effects of cultural ancestry and geographic proximity on pre-European Pacific forest outcomes, our analyses allow a re-evaluation of the putative predictors of deforestation and replacement, incorporating cultural factors in our model, controlling for spatial and phylogenetic non-independence in the data and using a rigorous model averaging approach. Taken together, our models of the most likely ecological and cultural predictors suggest that groups reliant on irrigated agricultural intensification and inhabiting low, dry, isolated sites that are beyond the range of volcanic ash fallout are particularly predisposed to greater levels of deforestation. Likewise, cultures with a reliance on arboriculture or elite land ownership and that inhabited small islands beyond the range of volcanic ash fallout are particularly predisposed to greater levels of forest replacement. These findings confirm the importance of ecology in shaping Pacific forest outcomes, but also highlight a role for cultural variation. Our cultural predictors were among the most important predictors of forest outcomes but, intriguingly, cultural ancestry, as measured by linguistic affiliations spanning many generations, appears not to constrain environmental impact.

The extent to which human development, resource management and social outcomes are shaped by the environment remains controversial [4,7,25–28]. Whilst ecological factors are argued by some to be key drivers of historical societal successes and failures [4,25,29–31], others label these conclusions ‘neo-environmental determinism’ for their underemphasis of the role of culture and oversimplification of more complex human-environment interactions [7,28]. The many islands of the Pacific afford a unique opportunity to move beyond these disagreements by investigating the importance of both ecological [3] and cultural factors [2,6] affecting human-environment interactions. Here, we were able to quantify these effects by explicitly incorporating both ecological and cultural predictors into a single analytical framework. Our findings highlight the role of cultural variation and challenge a strict environmental determinism, but also reveal that forest outcomes are not constrained by deep cultural ancestry. Hence, we find environmental impact reflects a combination of ecological constraints and the short term responses of each culture in the face of those constraints.

## Materials and Methods

### Data

Ecology and forest outcome data across 73 islands were sourced from ref [3]. Following ref [3], six islands without permanent pre-European settlements (Henderson, Pitcairn, Hatuta’a, Kahoolawe, Eua and Mohotani) were excluded from our analysis. Also following ref [3], 13 of the islands, on which rainfall differed substantially between leeward and windward sides, were divided into separate leeward and windward sites, producing a total of 80 sites (S1 File).

Pre-European forest outcome data were taken from ref [3], based on accounts of early European visitors. Deforestation scores range from 1 (no deforestation) to 5 (almost completely deforested) and are taken directly from ref. (1), but we used a modified version of the forest replacement measure ranging from 1 (<10% introduced species) to 4 (75–100% introduced species up to 600m). Ref [3] assigned a score of 5 to islands with no remaining forest cover, but we judged that islands with no remaining forest cannot be scored meaningfully because forest replacement is a measure of the proportion of existing forest comprising introduced species. We therefore removed the two islands with no trees from our main forest replacement analyses. See S11 Table for forest outcome coding scheme.

Ecological predictors—rainfall, elevation, area, isolation, absolute latitude, makatea coverage, age, volcanic ash fallout (tephra) and asian continental dust fallout—were taken from ref [3] and are summarized in S8 Table. Following ref [3], two variables (tephra and age) were treated as factors and four variables (rainfall, elevation, area, and isolation) were log transformed.

Land tenure system and mode of agricultural intensification were coded from ethnographic sources for each ethnolinguistic group in our language tree (see below). As far as possible, cultures were coded as they were at or around the time of early European contact. Agricultural intensification was coded as a reliance on wet, dry, arboricultural or no intensification, as outlined in ref [2] (S1 and S2 Tables). Land tenure was coded based on the presence or absence of individual or elite land ownership following [19] and [32] (S3 and S4 Tables).

### Language Tree-building

We used the Austronesian language family tree as a proxy for the cultural ancestry of groups at each location in our sample. To do this, we matched each site to the language or languages recorded at that site and for which vocabulary data was available in the *Austronesian Basic Vocabulary Database* (ABVD; [33]). This process resulted in some sites being assigned multiple languages. For example, the ABVD provided vocabulary data on five of the languages spoken on Santa Isabel in the Solomon Islands, so Santa Isabel was assigned language data for all five of these languages. In cases where one language from the ABVD was spoken at multiple sites, we added ‘dialects’ of this language at each site. For example, the language ‘Samoan’ is spoken at two of our sites, Savaii and Upolu. These two sites were assigned the dialects ‘Samoan1’ and ‘Samoan2’ respectively, and together they formed the group ‘Samoan’. In order to allow for dialect variation, and to integrate over uncertainty in the ancestral relationship between dialects, one dialect from each group was assigned vocabulary data (to locate the group appropriately on the tree) whilst its sister dialects were allowed to vary within constraints based on prior knowledge of their history of divergence (see S12 Table). The matching process resulted in sites being linked to a total of 119 languages/dialects.

Following [8], we inferred the relationship between the languages in our sample by coding vocabulary data from the ABVD and analysing it using Bayesian inference of phylogeny and Markov Chain Monte Carlo (MCMC) methods as implemented in *BEAST 1*.*7*.*5* [34]. The ABVD contains lists of 210 basic vocabulary terms for over half of the 1,200 Austronesian languages [33]. These terms signify universally meaningful concepts, are relatively temporally stable, and are resistant to intercultural transmission [33,35]. We model language change as the birth and death of cognates along lineages. Cognates are homologous words whose systematic sound correspondences indicate common ancestry. For example, the words for hand in Hawaiian and Tahitian are *lima* and *rima* respectively, and their words for skin are ‘*ili* and ‘*iri* respectively. The pattern of cognates across a sample of languages can be coded into a binary matrix in which 1 and 0 represent the presence or absence of each cognate set.

In line with previous work [8], we model the evolution of cognate presence/absence using a covarion model [36,37], which allows for cognates to transition from actively changing to non-changeable states, and a relaxed clock [38], which allows rates to vary across the tree. We ran three independent Markov chains for 20 million iterations, discarding the first 2 million iterations of each chain as burnin. This produced a posterior distribution of 54,000 trees. We used the *Tracer* tool in *BEAST* to examine the convergence of MCMC runs and the *TreeAnnotator* tool in *BEAST* to summarize trees in the form of maximum clade credibility tree (Fig 1). Whilst there can be some variation in the tree topology favoured by various types of linguistic data, our vocabulary based tree broadly reflects established groupings [8] and hence provides a proxy for cultural ancestry.

In order to ensure that our findings are not contingent on a specific Austronesian tree topology and language-site assignment, the results we report were averaged across 100 replicates, randomly selecting trees from the Bayesian posterior distribution. To avoid double-counting of societies where multiple languages were spoken, we randomly sampled languages from sites with multiple possible language assignments to maintain a maximum N of 80 sites across analyses. This allows us to integrate over uncertainty in the phylogenetic tree and language sampling process.

Three islands in our sample (New Britain, New Ireland and Bougainville) are also inhabited by non-Austronesian-speaking groups. Adding a dummy variable for the presence of non-Austronesian-speaking groups to our best fitting models of forest outcomes produced a poorer overall model fit (ΔAICc <2), identified no effect of non-Austronesian-speaking groups (p_deforestation_ = 0.781; p_replacement_ = 0.481) and did not affect parameters associated with our other predictors.

### Testing for phylogenetic signal

We quantify phylogenetic signal in forest outcome data and our predictor variables (S5 Table) using Pagel’s Lambda [23], as implemented in the *phylosig* function in the *Phytools* package [39] in the *R v*.*3*.*0*.*1* statistical package [40]. λ can vary between 0 and 1. A λ value of 1 indicates that the data fits a Brownian motion model of trait evolution along the branches of the phylogeny such that trait covariance is proportional to relatedness. A λ value that is zero or not significantly different from zero implies evolution independent of the phylogeny (no phylogenetic signal). All reported estimates are based on 100 replicates from our posterior distribution of langauge trees, sampling one language from each site.

### Phylogenetic Generalized Least-Squares analysis

We adapt a PGLS-spatial approach [16] to estimate the independent effects of our ecological and cultural predictors on forest outcomes, whilst simultaneously quantifying and controlling for non-independence in the data due to geographic proximity and shared cultural ancestry. This approach modifies the standard regression variance matrix (***V***), according to the formula:

V(λ,ϕ)=(1−ϕ)[(1−λ)h+λΣ]+ϕW

Where *λ* represents the size of the shared ancestry effect and *ϕ* represents the contribution of spaital effects. **Σ** is an *n x n* matrix comprising the shared path lengths on the phylogeny. This is proportional to the expected variances and covariances under a Brownian motion model of evolution along the branches of a phylogeny [23]. Here we compute 100 **Σ** matrices based on 100 replicates from our posterior distribution of Austronesian language trees. ***W*** is the spatial matrix comprising pairwise Great-circle distances between sites in our sample, calculated using the Haversine formula [41]. ***h*** is the diagonal of **Σ** representing the distance from the root to the each sampled language.

Here we report *λ*′ = (1 − *ϕ*)*λ*, the proportional contribution of phylogeny to variance, and *ϕ*, the proportional contribution of spatial effects to variance, after rewriting the above equation as:

V(λ,ϕ)=γh+λ′Σ+ϕW

where *γ* = (1 − *ϕ*)(1 − *λ*) is the proportion of variance independent of phylogeny and space [16].

First, we consider the effect of cultural ancestry and geographic proximity alongside the previously identified set of candidate ecological predictors of deforestation and forest replacement [3]. In their analysis of ecological predictors of forest outcomes, ref [3] found linear regression and robust regression gave identical results. Here, we use multiple regression within the PGLS framework to simultaneously estimate and control for the effect of cultural ancestry, geographic proximity and ecological predictors. To identify the most important predictors of Pacific forest outcomes, rather than the stepwise regression approach used previously [3], we evaluate all 1024 possible combinations of ecological predictors, using model averaging based on Akaike Information Criterion, with listwise deletion of missing data. Following [24], we calculate the relative variable importance (RVI) of each of the candidate ecological predictors as the sum of the corrected Akaike Information Criterion (AICc) weights of all the models including each variable. In order to test whether *λ* and *ϕ* significantly improved model fit, we used likelihood ratio tests to compare the likelihood of models fitting both *λ* and *ϕ*, only *λ*, only *ϕ*, or neither *λ* nor *ϕ*. In order to account for uncertainty in the Austronesian language phylogeny and language assignments, all results were averaged across the 100 replicates from our Bayesian posterior distribution of language trees, sampling one language from each site. Finally, we repeated the above model averaging procedure combining the best ecological predictors (RVI > 50%) of deforestation and replacement with agricultural intensification and land tenure cultural predictors.

As an indication of overall model fit, we provide R^2^ calculated using the formula:

$$R^{2}=1-SS_{reg}/SS_{tot}$$

Where *SS*_reg_ is the residual sum of squares in the PGLS fitted model accounting for spatial and phylogenetic non-independence, and SS_tot_ is the total sum of squares accounting for spatial and phylogenetic non-independence in a PGLS model with no predictors. We note that R^2^ values in a Generalized least squares framework are not comparable with those from ordinary least squares. In addition, because residuals are not orthogonal, it is difficult to partition variance across independent variable and so we do not provide partial r^2^ values. We do, however, provide r^2^ values from separate bivariate PGLS analyses predicting deforestation and forest replacement from a model including the intercept plus each ecological (S13 Table) and cultural (S9 Table) predictor.

## Supporting Information

S1 Fig Maps of Pacific forest outcomes data. Points are shaded according to Deforestation (top, red) and replacement (bottom, green) scores. Darker shading equates to increased deforestation/replacement.
(PDF)

S1 File Data table showing site data on forest outcomes, language assignment, location, ecological predictors and cultural predictors. (XLSX)

S1 Table Agricultural intensification coding scheme, adapted from Kirch (1994). (PDF)

S2 Table Agricultural Intensification coding. (PDF)

S3 Table Land Tenure coding scheme (adapted from Currie 2013 & Kushnick et al. 2014). (PDF)

S4 Table Land Tenure Norm coding. (PDF)

S5 Table Estimates of phylogenetic signal (lambda) for each of the ecological and cultural predictors, averaged across 100 replicates from the posterior distribution of language trees. (PDF)

S6 Table Ecological predictors of forest replacement including cultural ancestry (lambda) but not geographic proximity (phi). (PDF)

S7 Table Model of forest replacement including only cultural ancestry (lambda) and geographic proximity (phi). (PDF)

S8 Table Descriptions of ecological variables. (PDF)

S9 Table Table showing results predicting deforestation and forest replacement from separate models including the intercept plus each cultural predictor, and controlling for the effects of cultural ancestry and spatial proximity. (PDF)

S10 Table Ecological and cultural predictors of forest replacement (excluding Arboriculture). (PDF)

S11 Table Coding schemes for forest outcomes data based on descriptions from early European visitors. (PDF)

S12 Table Priors on divergence time of dialects and major clades imposed on Austronesian language phylogeny. (PDF)

S13 Table Table showing results predicting deforestation and forest replacement from separate models including the intercept plus each ecological predictor, and controlling for the effects of cultural ancestry and spatial proximity. (PDF)
