# Supplementary material for: Cultural and Environmental Predictors of Pre-European Deforestation on Pacific Islands
Source: PLoS One. 2016 May 27;11(5):e0156340. doi: 10.1371/journal.pone.0156340 (PMC4883741; doi:10.1371/journal.pone.0156340)
Supplement: S1 Fig — Points are shaded according to Deforestation (top, red) and replacement (bottom, green) scores. Darker shading equates to increased deforestation/replacement. (PDF) [file pone.0156340.s001.pdf]

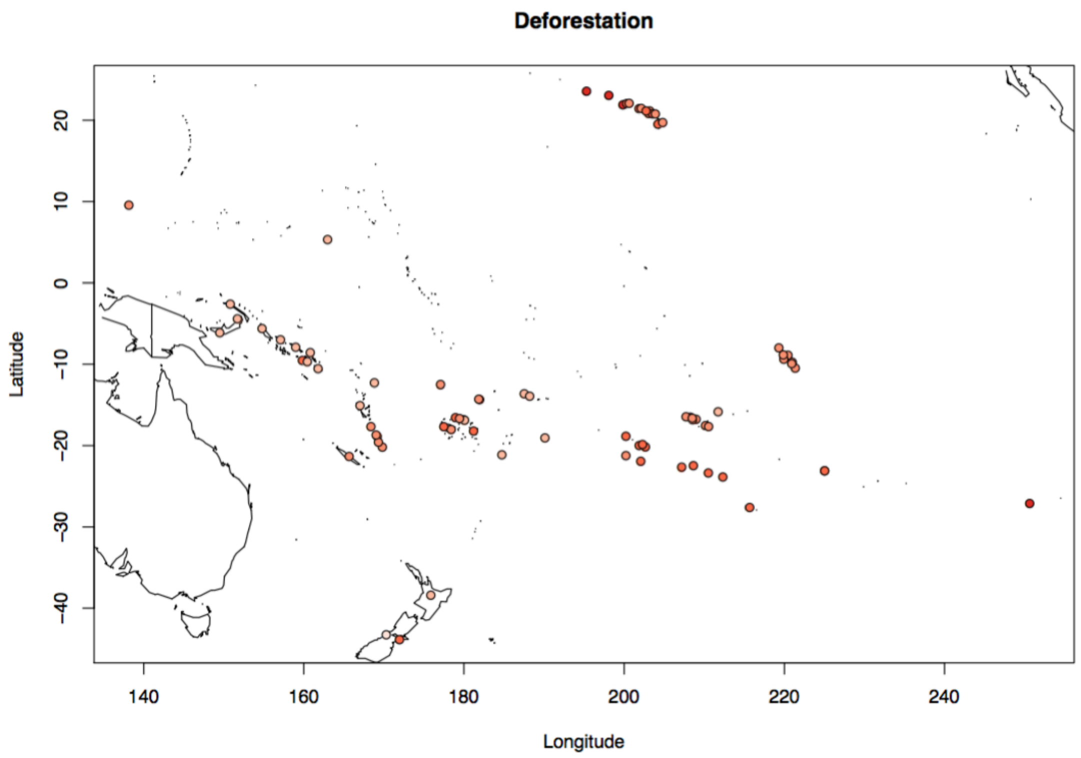

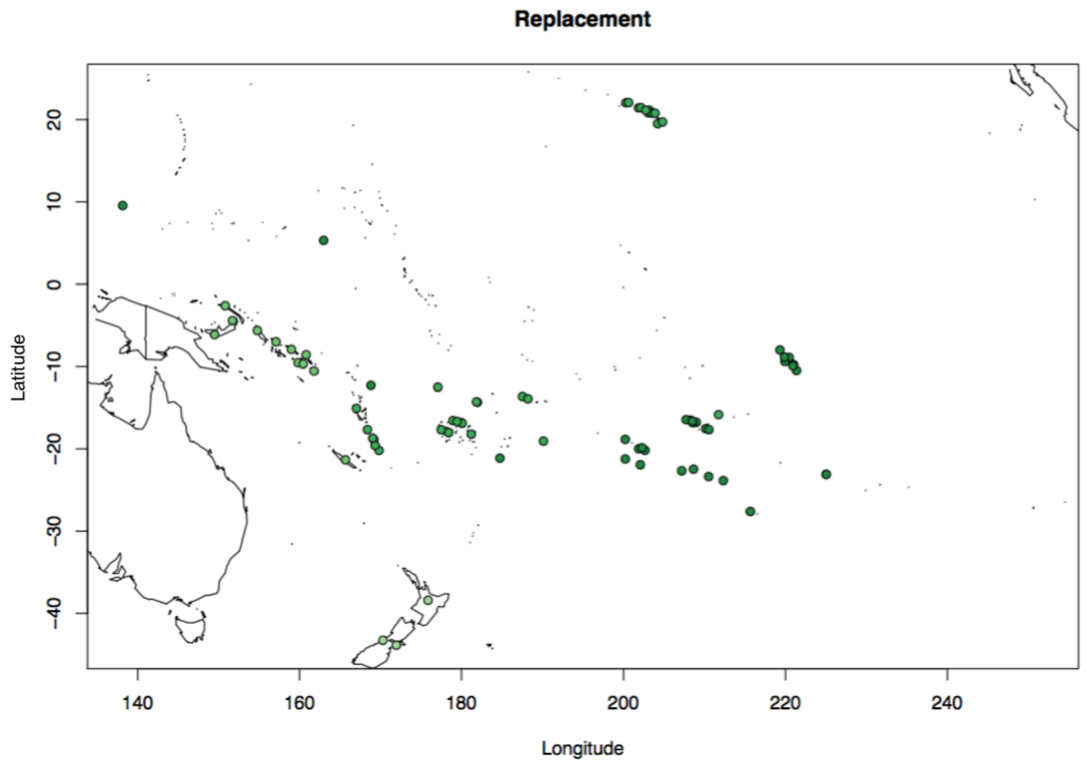

S1 Fig. Maps of Pacific forest outcomes data. Points are shaded according to Deforestation (top, red) and replacement (bottom, green) scores. Darker shading equates to increased deforestation/replacement.
