# Supplementary material for: Cultural and Environmental Predictors of Pre-European Deforestation on Pacific Islands
Source: PLoS One. 2016 May 27;11(5):e0156340. doi: 10.1371/journal.pone.0156340 (PMC4883741; doi:10.1371/journal.pone.0156340)
Supplement: S1 Table — (PDF) [file pone.0156340.s003.pdf]

S1 Table. Agricultural intensification coding scheme, adapted from Kirch (1994)			
**Code**	**Mode of intensification**	**Description**	**Example**
W	Wet	A reliance on water control to allow continuous cropping.	"The most important crop in the Cook Islands was taro, which was cultivated in wet field systems using techniques based either on irrigation or on drainage, according to the conditions of the local environment. In valleys with large enough catchments to support semi-permanent water flows, taro production was carried out in irrigated pondfield systems." (Walter, 1996, 74)
D	Dry	The use of techniques that shorten or eliminate fallow periods without relying on water control.	"In the westerly islands (Kauai, Oahu, Molokai and west Maui), the emphasis was on taro irrigation, with shifting cultivation and other forms of dryland gardening providing a distinctly secondary role. In the east (east Maui and Hawaii), irrigation was only a minor contributor to subsistence production and highly labor-intensive, short-fallow dryland field systems predominated". (Kirch, 1994, 253)
A	Arboriculture	Arboriculture associated with the long-term storage of tree crops.	"Aboriginally, subsistence was based on breadfruit, coconuts, bananas, taro, yams, and sugarcane. Breadfruit was the staple when in season. It was preserved in leaf-lined pits for times of scarcity." (Peoples, 1991, p 128).
N	No evidence of intensification	No evidence of wet, dry or arboricultural intensification as defined above.	"The ground is not very fertile, and after one crop of taro or yams has been grown, and perhaps one of sweet potatoes, a new patch of jungle has to be cleared." (Hogbin, 1939, pp 18-19)."
**Guidelines for agricultural intensification:**<br><br>If two or more forms of intensification are present within a given culture, the dominant form, i.e. the form that contributes the largest amount of energy to the diet, should be coded. If two or more forms are equally important, or if their relative importance is unclear, both forms (or all three forms if it is not possible to identify the two most important forms) should be coded (e.g. W, D or W, D, A).			

References

Hogbin, I. (1939). *Experiments in Civilization: The Effects of European Culture on a Native Community of the Solomon Islands*. London, England: George Routledge and Sons.

Kirch, P.V. (1994). *The Wet and the Dry: Irrigation and Agricultural Intensification in Polynesia.* Chicago, IL: University of Chicago Press.

Peoples, J.G. (1991). Kosrae. In T.E. Hays (Ed.), *Encyclopaedia of World Cultures (Volume II: Oceania)* (pp 128-131). New York, NY: G.K. Hall & Co.

Walter, R. (1996). Settlement Pattern Archaeology in the Southern Cook Islands: A Review. *The Journal of the Polynesian Society*, *105* (1), 63-99. Retrieved from http://www.jstor.org/stable/20706646
