# Supplementary material for: Cultural and Environmental Predictors of Pre-European Deforestation on Pacific Islands
Source: PLoS One. 2016 May 27;11(5):e0156340. doi: 10.1371/journal.pone.0156340 (PMC4883741; doi:10.1371/journal.pone.0156340)
Supplement: S2 Table — (PDF) [file pone.0156340.s004.pdf]

S2 Table. Agricultural Intensification coding.				
**Datapoint**	**Island**	**Language (s)**	**Coding Justification**	**Code**
Aneityum	Aneityum	Anejom	"The agricultural system consisted then – as it does in part today – of both dryland and irrigated gardens, with taro as the main staple, grown in different types of taro swamps and canal-fed, furrow-irrigated gardens. Several dryland swidden techniques were also practiced (see Spriggs 1981, chap 3)." (Spriggs, 2007, 282)	W
Austral Islands (Raivavae)	Raivavae	Rurutu	Kirch (1994B, p 6, Table 1)	W
Austral Islands (Rimarata)	Rimatara	Rurutu	Table 1 in Kirch (1994B, p 6) does not refer to Rimatara specifically, but does refer to three other islands of the Austral Group. Irrigation is described as the dominant method of intensification on all of the three.	W
Austral Islands (Rurutu)	Rurutu	Rurutu	Kirch (1994B, p 6, Table 1)	W
Austral Islands (Tubuai)	Tubuai	Rurutu	Kirch (1994B, p 6, Table 1)	W
Austral Islands (Rapa)	Rapa	Rapa	Kirch (1994B, p 6, Table 1)	W
Bougainville (Banoni)	Bougainville	Banoni	"Bougainvilleans were, and most continue to be, farmers … In pre-European days they obtained most of their foodstuffs from their own gardens, supplemented by fishing, pig raising, collection of tree crops and wild plants, and a little hunting … Taro gardens are laid out on well-drained terrain where the soil is deep and free of sand. Another technical requirement is that gardens be located in areas of secondary growth; I need not see a single instance of primary forest being	N
Bougainville (Taiof)		Taiof		
Bougainville (Teop)		Teop		
Bougainville (Torau)		Torau		

			cleared for gardens. They say that it is too difficult even with metal axes to cut down and remove the huge trees; but there are other reasons why new gardens are located on old garden sites … The ideal technique, in the north-east region, is to progress in one direction for about six years and then return to the starting point and begin again." (Oliver, 1973, pp 46-49).	
Choiseul (Babatana)	Choiseul	Babatana	"Since gardening techniques have undergone extensive change, I rely largely upon generalized accounts of past practices, but in a few 'bush' communities gardening is still practiced as I describe it, with important changes in the tools used … It is quite clear that taro production was the major subsistence concern in the past and that most other activities were adapted to that concern … The Choiseulese practice swidden horticulture because of their technology and the crops available to them … The land itself, because of rapid leaching of nutrients, is never suitable for more than one planting (the Choiseulese seem never to have used any form of irrigation) after which it must be left to fallow for varying periods. Ten to twenty or more years may be required, or such were the estimates of my informants … All of this means, of course, that large areas of land are required per capita and that local groups must have at their disposal much larger areas than they can use at any one time. The population must be dispersed and of relatively low density." (Scheffler, 1965, pp 10-11).	N
Choiseul (Ririo)		Ririo		
Choiseul (Vaghua)		Vaghua		
Choiseul (Varisi)		Varisi		
Cook Islands (Aitutaki)	Aitutaki	Rarotongan	"The most important crop in the Cook Islands was taro, which was cultivated in wet field systems using techniques based either on irrigation or on drainage, according to the conditions of the local environment. In valleys with large enough catchments to support semi-	W

			permanent water flows, taro production was carried out in irrigated pondfield systems. The most highly developed of these were on Mangaia, where Allen (1971) has recorded two major types of complex." (Walter, 1996, 74)<br><br>"The period of greatest change probably dates to between 3000 and 1200 BP, following a sea-level fall of 1 to 2 m. At this time offshore islets stabilized, coastal flats emerged, and marshy areas suitable for taro production developed." (Allen, 1997, 144-145)	
Cook Islands (Atiu)	Atiu	Rarotongan	"The most important crop in the Cook Islands was taro, which was cultivated in wet field systems using techniques based either on irrigation or on drainage, according to the conditions of the local environment … The most highly developed of these were on Mangaia, where Allen (1971) has recorded two major types of complex … Elsewhere on Rarotonga and Mangaia, as well as on Atiu and Ma'uke, drainage channels are also cut through the low standing swamps and the fill piled up to form raised planting beds. The function of the drainage systems is to channel excess water out of the planting zone rather than introduce it as in the irrigated pondfield systems. When not in use, drainage systems in swampy zones silt up very quickly but on all four of the above islands remnants of large drainage channels and beds can be seen adjacent to the still extensive areas under cultivation." (Walter, 1996, pp 74-77).	W
Cook Islands (Mangaia)	Mangaia		"While irrigated taro formed the core of Mangaian agriculture, both shifting cultivation and tree cropping on the makatea and on the lower colluvial slopes and ridge spurs provided other essential foods and industrial	W

			materials.” (Kirch, 1994B, 275)	
Cook Islands (Mau’ke)	Mau’ke		“The most important crop in the Cook Islands was taro, which was cultivated in wet field systems using techniques based either on irrigation or on drainage, according to the conditions of the local environment … The most highly developed of these were on Mangaia, where Allen (1971) has recorded two major types of complex … Elsewhere on Rarotonga and Mangaia, as well as on Atiu and Ma'uke, drainage channels are also cut through the low standing swamps and the fill piled up to form raised planting beds. The function of the drainage systems is to channel excess water out of the planting zone rather than introduce it as in the irrigated pondfield systems. When not in use, drainage systems in swampy zones silt up very quickly but on all four of the above islands remnants of large drainage channels and beds can be seen adjacent to the still extensive areas under cultivation.” (Walter, 1996, pp 74-77).	W
Cook Islands (Mitiaro)	Mitiaro		“The supply of vegetables was poor, as taro—the best vegetable of the tropics—does not grow on Mitiaro.”<br><br>(Gill, 1876, p 172)<br><br>“Other agricultural features recorded in Ngaputoru are stone boundary walls and pig pens. On all three islands, walls at least a kilometre in length with shorter side branches occur on the dryland soils along the inner margins of the makatea. Unlike some of the recorded systems on Hawai'i, these do not seem to demark specific plots and their exact function is unknown, although they are strongly associated with the dry agricultural soils. Small pig pens made of coral rubble are built just inside the inner edges of the makatea on all three islands of	D

			Ngaputoru. They are up to several metres in height and some have been sunk below surface level by using existing crevices in the upraised coral beds in which the entrances are blocked and walls heightened." (Walter, 1996, p 77).	
Cook Islands (Rarotonga)	Rarotonga		"The Rarotongan people were aware of the different soil types and their potentialities for various crops. Taro was planted in the valleys and the swampy depressions in the coastal flats. Most varieties were grown in swamps (both natural and artificial), and it was necessary to use a simple irrigation system of dams and water channels … It was probably due to the limited range of implements and to the fact that taro required very little clearing or other cultivation that it was the staple food … Shifting cultivation was practiced, but it applied only to the supplementary crops like sweet potatoes, arrowroots, yams and giant taro …" (Crocombe, 1964, p 19).<br><br>"The most important crop in the Cook Islands was taro, which was cultivated in wet field systems using techniques based either on irrigation or on drainage, according to the conditions of the local environment. In valleys with large enough catchments to support semi-permanent water flows, taro production was carried out in irrigated pondfield systems. The most highliy developed of these were on Mangaia, where Allen (1971) has recorded two major types of complex … Although smaller in scale, similar systems have been recorded on Rarotonga (Bellwood, 1978)." (Walter, 1996, 74)	W
Easter Island	Easter Island	Rapa Nui	"On Easter Island, large tracts were intensively cultivated, with an emphasis on the sweet potato. Metraux (1940:151-2) refers to the	D

			heavy use of grass mulch, as in Anuta and Hawaii. Unfortunately, the Easter Island dryland fields were not permanently delineated with stone rows or borders, and so have left no archaeological trace. Another form of intensive horticultural feature, the walled-garden plot or manavai, is however very much in evidence throughout the island (McCoy 1976).” (Kirch, 1984, 179)	
Efate	Efate	South Efate	Weightman (1989, pp 30-52). Excerpts:<br><br>“The wet northern and eastern central islands are mainly taro growing, particularly in their upland areas … The drier islands, such as Malakula, Ambrym, Paama, Epi, the Shepherds, Efate, Erromango, Tanna and Aniwa are principally yam-growing, but taro is again the most important food crop on Aneityum in the far south.” (Weightman, 1989, p 29).<br><br>“While [taro] is essentially a crop of the wet uplands of Santo, Ambae, Maewo and Pentecost, and the high rainfall islands of the Banks and Torres in the north, it is also commonly intercropped in annual high rainfall gardens, and grown under intensive irrigated systems in limited areas of Aneityum, Pentecost, Maewo, Vanua Lava and Santo… It is little grown on Paama, Malakula and Efate, and is practically non-existent in the Shepherds.” (Weightman, 1989, 88)<br><br>Weightman’s description of traditional Vanuatu cultivation systems (pp 30-52) suggests that intensified agriculture only occurred in the context of irrigation of taro, which appears not to have occurred	N

			on Efate. Dry agriculture seems to have been traditional long-fallow swiddening. At the end of the section, Weightman notes:<br><br>"While the continuous tectonic uplifting of Vanuatu, frequent earth tremors, and high rainfall and responsible for the cutting back of streams, erosion of steep hill land, and major landslides … there is little evidence, excepting on Aneityum, that man's activities have contributed significantly to soil erosion and degradation, despite the fact that considerable areas of steep land are cultivated. Again, it is the discontinuous nature of this cultivation, limited populations relative to arable land, and the small size of individual gardens and near-minimum tillage that have permitted these problems to be avoided." (Weightman, 1989, pp 51-52).	
Erromango (Sye)	Erromango	Sye	"HORTICULTURE. While the land belongs to the district group, gardens may be made by any man, in the bush, on land that has been deserted by a former garden-maker, and these are the sole property of the maker as long as he continues to care for them, but directly they are allowed to lie fallow they return to the district group and may be used by anyone at will. In practice, of course, no native will use a garden site that is lying fallow unless a sufficient time has elapsed to ensure a good harvest … The gardens produce yams, the principal food staple … taro … arrowroot …" (Humphreys, 1926, p 151).	N
Erromango (Ura)		Ura		
Espiritu Santo (Araki ((Southwest Santo))	Espiritu Santo	Araki (Southwest Santo)	Irrigated taro fields, some of which were extensive, occurred in parts of Santo:	W
Espiritu Santo (Sakao		Merei	"Irrigated fields are found only in the volcanic parts of the group: in	

(Merei))			western Santo, also in the region of the pygmies, in Pentecost, in Aoba, in Maevo, in the Banks Islands, and in Futuna and Aneitym (78:p.23;66, p. 59) … As a typical example I will describe a taro field with an irrigation system near Wora in northwest Santo, the largest I have ever seen. A stream with a fair volume of water flows through a narrow, rocky valley. At the point at which it changes direction, it has been dammed with large boulders, causing the water to be pent up. Above this dam a channel branches off … The channel runs along the valley side …” (Speiser, 1923 / 1990, p 134).<br><br>Weightman (1989, pp 30-52). Excerpt:<br><br>“While [taro] is essentially a crop of the wet uplands of Santo, Ambae, Maewo and Pentecost, and the high rainfall islands of the Banks and Torres in the north, it is also commonly intercropped in annual high rainfall gardens, and grown under intensive irrigated systems in limited areas of Aneityum, Pentecost, Maewo, Vanua Lava and Santo… It is little grown on Paama, Malakula and Efate, and is practically non-existent in the Shepherds.” (Weightman, 1989, 88)	
Espiritu Santo (Sakao (Port Olry))		Sakao (Port Olry)		
Fiji (Viti Levu (Lee))	Viti Levu (Lee)	Fijian (Bau)	Kuhlken (2002). Excerpts:<br><br>“In Fiji, while both yams and taro are significant in subsistence and ritual, it is the cultivation of several varieties of taro that has received the greatest measure and variety of intensity.” (Kuhlken, 2002, p 165)<br><br>“The cultivation of taro optimally	W, D
Fiji (Viti Levu (Wind))	Viti Levu (Wind)	Western Fijian (Navosa)		
Fijian (Lakeba)	Lakeba	Fijian		
Fijian (Taveuni	Taveuni	Fijian		

(Lee))	(Lee)		takes place within a wet ‘pondfield’ environment, provided either by a naturally swampy site or through irrigation technology.” (Kuhlken, 2002, p 167).<br><br>“Yam mounds are ephemeral agricultural landforms designed to optimize the microclimatic and edaphic environment favored by Dioscorea species. Eric Waddell (1972) produced the definitive explanatory work on these features, based on field research in Papua New Guinea; Wilken (1987) later listed a number of benefits from mounding, which include wind resistance, improved soil drainage and aeration, decrease in soil moisture losses from evaporation, and suppressed weed growth and facilitated weed control. Fijians took great care in cultivating yams because of their social and ritual significance. Lasaqa (1963, 54) gave us some indication of the status of this cultigen when he offered the following assessment, ‘The yam is traditionally the most important crop in Fiji. It was deeply involved in the social organization and in the general activities of the people. As such it had a high place in the Fijian agricultural calendar.<br><br>Growing yams takes up much time and labor, particularly as it requires the annual preparation of new gardens …" (Kuhlken, 2002, pp 169-170)<br><br>“Denevan and Turner (1974, 24) listed a number of Pacific island locations in their survey and discussion of tropical raised fields beyond the western hemisphere, and defined raised fields as ‘any prepared land involving the transfer and elevation of soil above the	
Fijian (Taveuni (Wind))	Tavenui (Wind)	Fijian		
Fijian (Vanua Levu (Lee))	Vanua Levu (Lee)	Fijian		
Fijian (Vanua Levu (Wind))	Vanua Levu (Wind)	Fijian		

			natural surface of the earth in order to improve cultivating conditions.' In Fiji, the general term for such features is vuci .... They are widespread throughout the islands, and on a small scale occur in most of the coastal plains and in the bottomlands of large river valleys. Vuci are commonly designed in such areas for the cultivation of dalo, or taro (Colocasia), by raising the planting surface above a saturated natural base … Most Fijian villages have at some point cultivated taro in raised fields, although certain areas exhibit more prevalent use of this intensive agricultural landform than others." (Kuhlken, 2002, p 172).<br><br>"The people of Fiji constructed irrigated terraces for the production of one specific cultigen—Colocasia esculenta, and taro terraces were once prevalent in many areas of the archipelago. Similar to that documented for raised fields, the widespread distribution of terracing is reflected in the different names given to these features in various parts of the country." (Kuhlken, 2002, pp 173-174).	
Futuna and Alofi (Alofi)	Alofi	Futuna, East	"In west Polynesia, Kirch (1975) observed that parts of Alofi Island the Horne group are characterised by abandoned dryland field systems. These are marked by field borders constructed of limestone cobbles, and presumably indicate former intensive cropping of yams and associated field crops." (Kirch, 1984, 179)<br><br>"Whereas Sigave could exploit the natural opportunities for pondfield irrigation of taro, Alo-Alofi was forced to follow a more 'burdensome' route of labor-intensive, short-fallow shifting cultivation, closer to the	D

			classic Boserup model of intensification." (Kirch, 1994B, p 244).	
Futuna and Alofi (Futuna)	Futuna	Futuna, East	"Futuna and Alofi were were particularly appealing as a field site because the two islands encompass a full spectrum of wet and dry agronomic environments, with associated variability in agricultural practice. The western half of Futuna, with its complex volcanic geological structure, offered many well-watered stream valleys with pondfield irrigation systems that were reported to be among the largest and most complex of anywhere in Polynesia. Alofi and most of Eastern Futuna, on the other hand, and composed of old, elevated limestone whose karst terrain prohibited irrigation but supported classic integral swidden cultivation regimes." (Kirch, 1994B, p 21).<br><br>"Whereas Sigave could exploit the natural opportunities for pondfield irrigation of taro, Alo-Alofi was forced to follow a more 'burdensome' route of labor-intensive, short-fallow shifting cultivation, closer to the classic Boserup model of intensification." (Kirch, 1994B, p 244).	W, D
Guadalcanal (Ghari)<br><br>Guadalcanal (Talise)	Guadalcanal	Ghari<br><br>Talise	"In his reviews of Solomon Islands agricultural systems Yen (1974b:40, 1976a:60) drew attention to the account of Catoira (Amherst and Thomson 1901:306) indicating the previous employment in Guadalcanal of more intensive agricultural practices than the rather uniform swidden systems now in general use… the Spaniards… *saw many villages upon the hills and many plantations of food on the slopes, arranged very well so that they could irrigate them, which they did. It was well laid out; and by each cleft there was a stream of water. The place*	W

<table>
<tr><td></td><td></td><td></td><td>whence the water came is full of trees.” (Roe, 1993, 159)

“Quite large-scale irrigated taro agriculture developed in suitable areas of north Guadalcanal.” (Spriggs, 1997, 174)</td><td></td></tr>
<tr><td>Hawaiian Islands (Hawaii (Lee))</td><td>Hawaii</td><td>Hawaiian</td><td rowspan="2">“The emphasis on dryland cultivation in Hawaii Island (and in Maui Island) was accompanied by a concomitant demand for intensive labour, and provides a significant contrast to the agricultural systems of the westerly Islands (Kauai, Oahu and Molokai), where taro irrigation dominated.” (Kirch, 1984, 189)

“In the westerly islands (Kauai, Oahu, Molokai and west Maui), the emphasis was on taro irrigation, with shifting cultivation and other forms of dryland gardening providing a distinctly secondary role. In the east (east Maui and Hawaii), irrigation was only a minor contributor to subsistence production and highly labor-intensive, short-fallow dryland field systems predominated.” (Kirch, 1994B, 253)</td><td rowspan="2">D</td></tr>
<tr><td>Hawaiian Islands (Hawaii (Wind))</td><td>Hawaii</td><td>Hawaiian</td></tr>
<tr><td>Hawaiian Islands (Kahoolawe)</td><td>Kahoolawe</td><td>Hawaiian</td><td>“Greater use of the inland plateau of Kaho'olawe for agriculture and reduction of fallow periods might have led to a change from a predominantly dry forest vegetation to a more open savannah, maintained by regular burning during the later prehistoric period. The burning would have encouraged growth of pili grass that might have been used as an agricultural mulch. Possible environmental effects of such a vegetation change have been discussed earlier but there is no clear evidence at present that vegetation clearance by European contact had rendered the island increasingly marginal for agriculture. The historic sources already quoted from the 1850s state that at that time sweet potatoes, melons, pumpkins, and</td><td>D</td></tr>
</table>

			sugarcane were growing well on the inland plateau (Allen 1858; Nahaolelua and Richardson 1857).” (Spriggs, 1991, p 101).	
Hawaiian Islands (Kauai- Lee)	Kauai (Lee)	Hawaiian	“In the westerly islands (Kauai, Oahu, Molokai and west Maui), the emphasis was on taro irrigation, with shifting cultivation and other forms of dryland gardening providing a distinctly secondary role. In the east (east Maui and Hawaii), irrigation was only a minor contributor to subsistence production and highly labor-intensive, short-fallow dryland field systems predominated.” (Kirch, 1994B, 253)	W
Hawaiian Islands (Kauai – Wind)	Kauai (Wind)			
Hawaiian Islands (Lanai)	Lanai	Hawaiian	“Slight terraces, small enclosures, and cleared lanes for yams, sweet potatoes, gourds, and the introduced water-melon, are found near all house sites. Cleared patches of land are sometimes attached to the house platforms and sometimes detached. None of the enclosing stone walls are high enough to keep out pigs … Terraces for taro are also seen at the head of Manalei gulch. On either side of the stream are a number of stone-faced terraces …” (Emory, 1924, p 48)<br><br>“Major zones are absent on the lower islands of Molokai, Lanai and Niihau where rainfall and catchment sizes are both limited.” (Earle, 1980, 9)	D
Hawaiian Islands (Maui (Lee))	Maui (Lee)		“In the westerly islands (Kauai, Oahu, Molokai and west Maui), the emphasis was on taro irrigation, with shifting cultivation and other forms of dryland gardening providing a distinctly secondary role. In the east (east Maui and Hawaii), irrigation was only a minor contributor to subsistence production and highly labor-intensive, short-fallow dryland field systems predominated.” (Kirch, 1994B, 253)	W
Hawaiian Islands (Maui	Maui (Wind)		“In the westerly islands (Kauai, Oahu, Molokai and west Maui), the	D

(Wind))			emphasis was on taro irrigation, with shifting cultivation and other forms of dryland gardening providing a distinctly secondary role. In the east (east Maui and Hawaii), irrigation was only a minor contributor to subsistence production and highly labor-intensive, short-fallow dryland field systems predominated." (Kirch, 1994B, 253)	
Hawaiian Islands (Molokai (Lee))	Molokai (Lee)	Hawaiian	"In the westerly islands (Kauai, Oahu, Molokai and west Maui), the emphasis was on taro irrigation, with shifting cultivation and other forms of dryland gardening providing a distinctly secondary role. In the east (east Maui and Hawaii), irrigation was only a minor contributor to subsistence production and highly labor-intensive, short-fallow dryland field systems predominated." (Kirch, 1994B, 253)	W
Hawaiian Islands (Molokai (Wind))	Molokai (Wind)			
Hawaiian Islands (Necker)	Necker	Hawaiian	"In the westerly islands (Kauai, Oahu, Molokai and west Maui), the emphasis was on taro irrigation, with shifting cultivation and other forms of dryland gardening providing a distinctly secondary role." (Kirch, 1994B, 253)<br><br>"The dryland agricultural terraces and water diversion or catchment features (on Necker and Nihoa) are constructed using the same dry-laid masonry techniques present throughout Hawaii." (Lebo, 2004, 40)	W
Hawaiian Islands (Nihoa)	Nihoa	Hawaiian	"In the westerly islands (Kauai, Oahu, Molokai and west Maui), the emphasis was on taro irrigation, with shifting cultivation and other forms of dryland gardening providing a distinctly secondary role." (Kirch, 1994B, 253)<br><br>"The dryland agricultural terraces and water diversion or catchment features (on Necker and Nihoa) are constructed using the same dry-laid	W

			masonry techniques present throughout Hawaii.” (Lebo, 2004, 40)	
Hawaiian Islands (Niihau)	Niihau	Hawaiian	“With its limited rainfall, Ni'ihau's crop production was miniscule compared with the other Hawai’ian islands, excepting Kaho'olawe … Ni'ihau once produced large, good tasting yams. Preferred over sweet potatoes because they keep much longer at sea, yams put Ni'ihau on the navigational charts of many a ship's captain. Although Ni'ihau's terrain is barren and relatively inhospitable, yams were ‘planted presumably in the large pockets of elevated coral at the southeast of the island, the bottoms of which are said to be filled with vegetable mold’ (Handy and Handy 1972:434).” (Stepien, 1988, pp 15-16).	D
Hawaiian Islands (Oahu (Lee))	Oahu (Lee)	Hawaiian	“In the westerly islands (Kauai, Oahu, Molokai and west Maui), the emphasis was on taro irrigation, with shifting cultivation and other forms of dryland gardening providing a distinctly secondary role. In the east (east Maui and Hawaii), irrigation was only a minor contributor to subsistence production and highly labor-intensive, short-fallow dryland field systems predominated”. (Kirch, 1994B, 253)	W
Hawaiian Islands (Oahu (Wind))	Oahu (Wind)			
Henderson Island	Henderson	NA	NA	NA
Kosrae	Kosrae	Kusaie	“Aboriginally, subsistence was based on breadfruit, coconuts, bananas, taro, yams, and sugarcane. Breadfruit was the staple when in season. It was preserved in leaf-lined pits for times of scarcity.” (Peoples, 1991, p 128).	A
Malaita (Lau)	Malaita	Lau	“Like other peoples of the Malaita mainland, Kwara’ae are a ‘bush people’ … who produce most of their own food in gardens cleared from the forest and meet many of their other needs from the forest itself. As in other tropical forest environments,	N
Malaita (Lau (North))		Lau (North)		
Malaita (Lau (Walade)		Lau (Walade)		

Malaita (Saa)		Saa	the land is fertile enough to bear only one or two crops before it has to be left for the forest to regrow and replenish the soil. In the past most of Kwara’ae was covered with dense secondary forest recovering from or waiting for cultivation, broken only by clearings for temporary gardens and settlements. If gardens are short-lived, many settlements are scarcely more permanent …” (Burt, 1994, p 23).<br><br>"The principal crop is taro, but sweet potatoes and bananas are also grown, as well as a few yams ... Garden work occupies the greater portion of the natives' time, for they have to grow large quantities of produce not only for their own immediate use, but also for exchange with the saltwater people. They cultivate the ground, in fact, to provide themselves with fish as well as vegetables ... The ground is not very fertile, and after one crop of taro or yams has been grown, and perhaps one of sweet potatoes, a new patch of jungle has to be cleared." (Hogbin, 1939, pp 18-19).	
Malaita (Areare (Waiahaa Village))		Areare (Waiahaa Village)		
Malaita (Areare (Maasupa Village)		Areare (Maasupa Village)		
Mangareva (Lee)	Mangareva (Lee)	Mangareva	Buck (1938, pp 202-210). Excerpt:<br><br>“Breadfruit was the most valued vegetable food and, fermented, was the staple food with fish.” (Buck, 1938, p 202).<br><br>“Principal tree crops include coconut, breadfruit, taihitian chestnut, candlenut and vi apple … However, it seems that taro was an equally important food, raised wherever possible in small spring-fed irrigated pondfield systems situated in the valley bottoms.” (Kirch, 2004, pp 22-29)	A, W
Mangareva (Wind)	Mangareva (Wind)			

Marquesas (Eiao)	Eiao	Marquesan	Kirch (1984, p 171, Table 19).<br><br>“Where sufficient running water was available, wet taro cultivation was practiced, but dry taro plantings were probably more common … Wet or dry, taro was grown in sufficient quantity for A.J. von Krusenstern to note its presence in the plantations surrounding dwellings … While taro and other root crops required constant replanting to maintain continuous production, this was not true of the prime staples preferred by the Marquesans: breadfruit, coconuts, and bananas … it was breadfruit, with its ability (through fermentation) to be stored for extended periods of time, that was of prime importance to the culture. So vital was it that if for any reason a man or woman was heard to curse the tree or its fruit, nothing could save the person from an ordained death except flight to another island.” (Ferdon, 1993, pp 87-89).	A
Marquesas (Fatu Hiva)	Fatu Hiva	Marquesan		
Marquesas (Hatuta’a)	Hatuta’a	Marquesan		
Marquesas (Hiva Oa)	Hiva Oa	Marquesan		
Marquesas (Nukuhiva)	Nukuhiva	Marquesan		
Marquesas (Tahuata)	Tahuata	Marquesan		
Marquesas (Ua Huka)	Ua Huka	Marquesan		
Marquesas (Ua Pou)	Ua Pou	Marquesan		
New Britain (Kuanua)	New Britain	Kuanua	Brookfield and Hart (1971, p 117). New Britain placed in lowest class of agricultural intensity:<br><br>“This class covers over three quarters of all the places listed, and indeed occupies most of the area of Melanesia, with about half the total population. Broadly speaking all these places employ an essentially ‘swidden’ type of agriculture, depending on prolonged natural regeneration between periods of cultivation.” (Brookfield and Hart, 1971, p 105)<br><br>Rolett (2008) makes a similar point:<br><br>“Most western Pacific Islands,	N
New Britain (Lunga Lunga (Minigir))		Lunga Lunga (Minigir)		
New Britain (Maututu)		Maututu		
New Britain (Nakanai (Bileki Dialect))		Nakanai (Bileki Dialect)		
New Britain (Amara)		Amara		
New Britain (Kove)		Kove		
New Britain (Aria)		Aria		
New Britain		Kaulong (Au		

(Kaulong (Au Village))		Village)	including the Bismarck and Solomon archipelagoes, are large and mountainous … Shifting cultivation of rainfed gardens has remained the dominant strategy since the beginning of agriculture in this region, with arboriculture as a secondary component of food production systems. Despite 30,000 years or more of human settlement and a reliance on shifting cultivation for at least 4000 years, the Bismarcks and the Solomons remained densely forested until the advent of modern clear-cutting.” (Rolett, 2008, p 5)	
New Britain (Sengseng)		Sengseng		
New Britain (Bilur)		Bilur		
New Caledonia (Canala)	New Caledonia	Canala	“The last 700 years or so in New Caledonia saw a … marked intensification of wetland and dryland agriculture.” (Spriggs, 1997, 219)<br><br>“When we take into account the additional production of the extensive dryland field systems of the plains and the hills of Paita, which cover more than 10,000 hectares, as well as the potential input from fishing and shellfish gathering … one cannot avoid the conclusion that – even disregarding the question of surplus production – the size of the Kanak population at the end of the prehistoric period of these 30 km of coastal plains simply had to have been substantially more than the mere 900 people proposed by ethnographic accounts (Sand, 1995, 218-231).” (Sand, Bole and Ouetcho, 2007, p 315)<br><br>The artificial irrigation systems on Grande Terre have been identified as the most complex of Oceania (Kirch & Lepofsky 1993). Their extensive distribution over the island, compared to the other high islands of Melanesia including Fiji, must have been in respose to the cycles of	W, D
New Caledonia (Jawe)		Jawe		
New Caledonia (Nelemwa)		Nelemwa		

			drought that the archipelago experiences on a regular basis.” (Sand, Bole and Ouetcho, 2007, pp 315-316)	
New Ireland (Tigak)	New Ireland	Tigak	Brookfield and Hart (1971, p 117). New Britain placed in lowest class of agricultural intensity:<br><br>“This class covers over three quarters of all the places listed, and indeed occupies most of the area of Melanesia, with about half the total population. Broadly speaking all these places employ an essentially ‘swidden’ type of agriculture, depending on prolonged natural regeneration between periods of cultivation.” (Brookfield and Hart, 1971, p 105).<br><br>Rolett (2008) makes a similar point:<br><br>“Most western Pacific Islands, including the Bismarck and Solomon archipelagoes, are large and mountainous … Shifting cultivation of rainfed gardens has remained the dominant strategy since the beginning of agriculture in this region, with arboriculture as a secondary component of food production systems. Despite 30,000 years or more of human settlement and a reliance on shifting cultivation for at least 4000 years, the Bismarcks and the Solomons remained densely forested until the advent of modern clear-cutting.” (Rolett, 2008, p 5).	N
New Ireland (Kara (West))		Kara West,		
New Ireland (Nalik)		Nalik		
New Ireland (Patpatar)		Patpatar		
New Ireland (Kandas)		Kandas		
New Zealand (North Island)	North Island	Maori	“While the Anutan example is perhaps the only surviving ethnographic instance of intensive dryfield cultivation in Polynesia, recent archaeological work has revealed that similar systems were formerly operative in various island	D

			groups, In New Zealand, work by H. Leach (1979), Sullivan (1972, 1981), Lawlor (1980, 1981a, b) and others has documented a range of intensive walled-garden complexes throughout various parts of North Island. These field-field cropping complexes focused on the cultivation of sweet potato … At Puhinui, which radiocarbon dates have shown to be intensively cultivates in the sixteenth century AD (Lawlor 1981b), the dominant agronomic features are linear 'stone rows', and stone mounds and heaps…" (Kirch, 1984, 178)	
New Zealand (South Island (East Coast))	South Island (East Coast)	South Island Maori	"The response of the Maori agriculturists to the deteriorating conditions would have been horticultural in nature - an attempt to modify the environment to fit the plant's requirements for survival. The modern parallel to this is the use of glasshouses for non-adapted tropical or out-of-season plants. The Maori was more limited in what he could achieve, but the erection of wooden fences for the diversion of cold winds and the use of gravel on growing kumara plants for heat retention are measures of this type recorded. The stone walls which occur in most of New Zealand have been related to Maori kumara growing … South Island kumara growing is reasonably well established in earlier writings, e.g., in Canterbury, Nelson, Marlborough, and the southern-most limit at Temuka, South Canterbury is set on the discovery of pits construed to have been for storage." (Yen, 1961, pp 342-343)	D
New Zealand (South Island (West Coast))	South Island (West Coast)			
Niue	Niue	Niue	"The cultivations (mala) of the people are generally situated away from the villages, often considerable distances. They are usually in newly-cleared land, and it is through this process of clearing that a good deal of the island is now in scrub, or a second growth of wood." (Smith,1983, p 8)	N

			"There are no swamps or creeks or standing water sources (except for ponds in some caves) and soils are of poor nutrient status. Agriculture, consequently, has remained to this day a swiddening system." (Walter and Anderson, 1995, 473)	
Pitcairn	Pitcairn	NA	NA	NA
Rotuma	Rotuma	Rotuman	"Shifting cultivation, on a relatively short rotation, is practised with the result that most of the so-called forest is essentially second-growth. Hill tops are nearly always used for the cultivation of certain species of taro (dalo) … Mulching is undertaken with the fallen leaves from the cut trees. As a result of high rainfall, leaching is great, but it is compensated for by the large amount of decomposed vegetable matter." (Fatiaki, 1977, pp 1-2).	D
Samoa (Savaii)	Savaii	Samoan	Watters (1958). Excerpts:<br><br>"Prodigal use of the land in Old Samoa took the form of a system of shifting cultivation, rather less thorough in type than occurs in many tropical countries … Six staple vegetable foods monopolized the Samoan diet - taro, yam, ta'amu, coconut, breadfruit, and banana. The complete forest cover had to be cleared to establish groves for the tree crops and ground for the tubers." (Watters, 1958, p 340).<br><br>"Although taro (Colocasia esculentum) was the most important of all vegetable crops, yams (Dioscorea affinis alata) were often planted as the first crop of the new clearing … A loose soil structure was necessary to permit maximum tuber growth, and free drainage essential to preclude waterlogging of the root zone. As the yams grew, mounds	D
Samoa (Upolu)	Upolu			

			were raised about them, a practice that ensures growth in the loose top soil, rich in organic matter." (Watters, 1958, p 341).<br><br>"A food more easily cultivated and esteemed more highly was taro, which invariably followed yam cultivation, but which was also often the pioneer crop of virgin land. This crop occupied about 70 per cent of Samoan gardens … A mulch of dead vegetation was spread over the ground between plants to keep down weeds. About two weedings seem to have been done, but only just around each taro hole. The competition of weeds was harmful, but weeds helped to keep overdrained soils moist, and on steep slopes a matting of weeds limited soil erosion." (Watters, 1958, p 342).<br><br>"Taro was occasionally grown in swamps, although this was much less common than dry culture and the tubers were smaller … Several early visitors believed that the neat taro ponds were artificial, and that Samoans had developed a system of irrigation like other Polynesians. In fact no irrigation was done; the ponds were natural and the channels were drains." (Watters, 1958, p 343).	
San Cristobal (Arosi)	San Cristobal	Arosi	Brookfield and Hart (1971, p 117). San Cristobal placed in lowest class of agricultural intensity:<br><br>"This class covers over three quarters of all the places listed, and indeed occupies most of the area of Melanesia, with about half the total population. Broadly speaking all these places employ an essentially 'swidden' type of agriculture, depending on prolonged natural regeneration between periods of	N
San Cristobal (Fagani)		Fagani		
San Cristobal (Bauro (Haununu))		Bauro (Haununu)		
San Cristobal (Kahua)		Kahua		
San Cristobal		Bauro		

(Bauro (Pawa Village))		(Pawa Village)	cultivation." (Brookfield and Hart, 1971, p 105).<br><br>This is consistent with Fazey et al (2011):<br><br>"Participants also frequently reported changes to cultivated capital, such as reductions in yields of food crops. This was partly described as being the result of diseases and lack of space for food gardens, which resulted in reductions in fallow periods (in the most extremes cases, from a traditional 7-14 years to less than one year)." (Fazey et al., 2011, 1280)	
San Cristobal (Bauro (Baroo Village))		Bauro (Baroo Village)		
Santa Isabel (Samasodu)	Santa Isabel	Laghu (Samasodu)	Brookfield and Hart (1971, p 117). New Britain placed in lowest class of agricultural intensity:<br><br>"This class covers over three quarters of all the places listed, and indeed occupies most of the area of Melanesia, with about half the total population. Broadly speaking all these places employ an essentially 'swidden' type of agriculture, depending on prolonged natural regeneration between periods of cultivation." (Brookfield and Hart, 1971, p 105).<br><br>This is consistent with Bogesi (1948):<br><br>"Gardening. For removing the bark of trees stone axes called mahavu are used. The trees are then left to dry. Small bushes are brushed by sticks and left to dry, and ropes of split bamboo are cut. After the bushes are dry they are burned. This process is called sabo, and the heaping up of the rubbish in piles, punui. This is all men's work. The garden is measured	N
Santa Isabel (Zabana (Kia))		Zabana (Kia)		
Santa Isabel (Kokota)		Kokota		
Santa Isabel (Blabanga)		Blablanga		
Santa Isabel (Cheke Holo)		Cheke Holo		

			out (nohi) by laying sticks or marking the ground with sticks. Then the day for planting (jou) is selected. Taro tops are then carried over by women and left in the garden. Next, food must be prepared. Men and women of the entire village gather for the planting. The men take a stick sharpened at one end called supale. The men bore holes with the supale, and the women plant taro tops in the hole. The whole garden is planted in a day, with much humour, shouting and laughing, to the enjoyment of all." (Bogesi, 1948, 220)	
Society Islands (Bora Bora)	Bora Bora	Tahitian (Modern)	Lepofsky (1999). Excerpts:<br><br>"Short-fallow swiddens are those in which fallow lengths are not sufficiently long for the successional vegetation to approach the primary forest in age or structure. In short-fallow systems, plot fertility and productivity decline with continual use. To maintain or increase plot productivity, greater labor per plot is required. This intensification of labor may be in the form of mulching, mounding, intercropping, or terracing of sloped terrain (e.g., Yen 1973… Ethnohistoric accounts suggest that shifting cultivation was the primary means of cultivation in the inland portions of the high islands of the Society Islands." (Lepofsky, 1999, p 15).<br><br>"Contrary to previous summaries, my reading of the ethnohistoric sources is that wetfield cultivation of aroids, primarily taro (Colocasia esculenta), was a major component of Maohi cultivation systems. Two kinds of wetfield cultivation systems were used in the Society Islands: true irrigation and swamp cultivation (see Damm 1951; Spriggs 1981, 1984)." (Lepofsky, 1999, p 19).	W, A, D
Society Islands (Huahine)	Huahine	Tahitian (Modern)		
Society Islands (Maupiti)	Maupiti	Tahitian (Modern)		
Society Islands (Moorea)	Moorea	Tahitian (Modern)		
Society Islands (Raiatea)	Raiatea	Tahitian (Modern)		
Society Islands (Tahaa)	Tahaa	Tahitian (Modern)		
Society Islands (Tahiti)	Tahiti	Tahitian (Modern)		

			"Tree crops were grown by the Maohi both in large expansive zones dominated only by arboriculture, as well as in the upper canopy of house gardens. However, the role of the former in the Maohi cultivation system may have been somewhat exaggerated in previous summaries of Maohi cultivation (e.g., Oliver 1974; Lethwaite 1964). Several Europeans note extensive tree-crop zones along the coast …It is difficult to know how to interpret these and similar accounts (e.g., Robertson I955: 24), as they are based on impressions from sea. From that perspective, the tree-cropping component of cultivation would have dominated the landscape, while the other cultigens would have been invisible … Despite these biases, there is little doubt that expansive zones of tree crops were cultivated throughout the landscape." (Lepofsky, 1999, p 13-14)	
Tanna (Kwamera)<br>Tanna (Lenakel)<br>Tanna (Tanna Southwest)	Tanna	Kwamera<br>Lenakel<br>Tanna Southwest	"At the present time, with the reduced though still large population, the rotation of garden sites for all is possible, with the result that the crops are abundant … After two or three years' use garden sites are allowed to lie fallow for some years … The crops are planted in rotation. The chief ones on Tanna are Taro, manioc, sweet potato, sugar, some maize and, last but not least, the yam." (Humphreys, 1926, p 62).<br><br>"Given the topography and water resources of Tanna, irrigation systems for year-round production of taro could not have been developed… On Tanna, however, there is fertile land for dry land agriculture, particularly in more leeward areas. Thus instead of taro it was the seasonal dry land yam crop on which attention was focused." (Spriggs, 1986, 17)	D

Tikopia	Tikopia	Tikopia	"Despite such trends in common, the Tikopia – with their distinctive arboricultural mode of intensification – created an 'endpoint' agricultural landscape … Through a particular combination of historical contingency, human choice, and environmental constaint, the Tikopia gradually evolved a highly intensive, multi-storey system of orchard gardening … Technological innovations such as the use of masi fermentation and storage to confer temporal stability to food distribution were also critical components of this arboricultural strategy." (Kirch, 1994B, pp 291-292).<br><br>"In terms of relative amounts consumed, the staple vegetable food is the taro, termed by the natives themselves 'the basis of food'. Secondary to it in importance are the breadfruit (known metaphorically as te urufenua, the 'head of the land'), banana, and coco-nut, the last sometimes being used alone and sometimes in conjunction with the others … Taro matures in four or five months, and can yield a crop at any time of the year … The fresh 'seedlings' planted are the tops removed from the mature corms used for food, so that harvest and planting are intimately connected. The soil is prepared by cutting the brushwood, a process termed autaru, and then breaking up the ground to a depth of about nine inches with the digging stick … As the digging proceeds the tops of the brushwood are piled on one side and then laid over the dug earth. This process is known as ufiufi ('covering')." (Firth, 1939, pp 64-66)	A, D

Tonga (Tongatapu)	Tongatapu	Tongan	"At European contact, the agricultural system consisted of a short-fallow swidden system, in which yams (especially *Dioscorea alata)*, aroids (especially *Alocasia macrorrhiza*, but also *Colocasia esculenta*), and bananas were the principal field crops …Two aspects of this intensive dryland agriculture are of note (1) the shortness of the fallow period; and (2) the stress on permanent land division." (Kirch, 1984, p 221)	D
Makatea	Makatea	Tuamotu	"The Tuamotu archipelago extends from 14° to 25°S. latitude and from 125° to 149°W. longitude … Except for Makatea and one or two other raised coral islands, the Tuamotus are all low-lying atolls … The soil of these islands, made up of broken-down coral and a little leaf mold, is too sterile for breadfruit, banana, or citrus trees, and there is not enough water for the cultivation of taro. Coconuts and pandanus fruits therefore supply the bulk of the vegetable diet, which is supplemented by the boundless quantities of fish and shellfish found in the lagoons and adjacent sea." (Weckler, 1943, pp 76-77)	N
Yap	Yap	Yapese	"Subsistence on Yap is heavily reliant on agriculture. Aroids, yams, breadfruit, bananas, Polynesian chestnuts, cassava and coconuts are grown, but the major cultivated crop is giant swamp taro (*Cyrtosperma chamissonis*). The latter is grown in swamp ponds, either at the base of gradual slopes within village proper zones, or as large expanses of human constructed swamps along the coast. These landscape modifications probably developed in parallel with the stratified social system." (Dodson & Intoh, 1999, p 18)	W

References

Allen, M.S. (1997). Coastal Morphogenesis, Climatic Trends and Cook Islands Prehistory. In in P.V. Kirch, & T.L. Hunt, (Eds.), *Historical Ecology in the Pacific Islands, Prehistoric Environmental and Landscape Change* (pp 124-146). New Haven, CT: Yale University Press.

Bellwood, P.S. (1971). Varieties of Ecological Adaptation in the Southern Cook Islands. *Archaeology and Physical Anthropology in Oceania*, *6* (2), 145-169. Retrieved from http://www.jstor.org/stable/40386143

Bogesi, G. (1948). Santa Isabel, Solomon Islands (Continued). *Oceania*, *18* (4), 327-357. Retrieved from http://www.jstor.org.ezproxy.auckland.ac.nz/stable/40328178

Brookfield, H.C & Hart, D. (1971). *Melanesia: A Geographical Interpretation of an Island World.* London, England: Methuen and Co.

Buck, P.H. (1938). *Ethnology of Mangareva*. Honolulu, HI: The Museum.

Burt, B. (1994). *Tradition and Christianity: the Colonial Transformation of a Solomon Islands Society.* Chur, Switzerland: Harwood Academic Publishers.

Crocombe, R.G. (1964). *Land Tenure in the Cook Islands.* Melbourne, Australia: Oxford University Press.

Dodson, J.R. & Intoh, M. (1999). Prehistory and Palaeoecology of Yap, Federated States of Micronesia. *Quaternary International*, *59* (1), 17-26. DOI:10.1016/S1040-6182(98)00068-8

Earle, T. (1980). Prehistoric Irrigation in the Hawaiian Islands: An Evaluation of Evolutionary Significance. *Archaeology & Physical Anthropology in Oceania*, Vol. 15, No. 1 (Apr., 1980), pp 1-28. Retrieved from http://www.jstor.org/stable/40386400

Emory, K.P. (1924). *The Island of Lanai: A Survey of Native Culture.* Honolulu, HI: The Museum.

Epstein, A.L. (1991). Tolai. In T.E. Hays, (Ed.), *Encyclopaedia of World Cultures (Volume II: Oceania)* (pp 333-336). New York, NY: G.K. Hall & Co.

Fatiaki, A. (1977). An Introduction and Geography. In C. Plant, (Ed.), *Rotuma: Split Island* (pp 1-6). Suva, Fiji: University of the South Pacific.

Fazey, I., Pettorelli, N., Kenter, J., Wagatora, D., & Schuett, D. (2011). Maladaptive Trajectories of Change in Makira, Solomon Islands. *Global Environmental Change*, 21(4), 1275-1289. DOI: 10.1016/j.gloenvcha.2011.07.006

Ferdon, E.N. (1993). *Early Observations of Marquesan Culture, 1595-1813*. Tucson, AZ: University of Arizona Press.

Firth, R. (1939). *Primitive Polynesian Economy*. London, England: Routledge and Sons.

Gill, W.W. (1876). *Life in the Southern Isles*. London, England: The Religious Tract Society.

Hogbin, I. (1939). *Experiments in Civilization: The Effects of European Culture on a Native Community of the Solomon Islands*. London, England: George Routledge and Sons.

Humphreys, C.B. (1926). *The Southern New Hebrides: An Ethnological Record*. Cambridge, England: Cambridge University Press.

Kirch, P.V. (1984). *The Evolution of the Polynesian Chiefdoms*. Cambridge, England: Cambridge University Press.

Kirch, P.V. (1994A). The Pre-Christian Ritual Cycle of Futuna, Western Polynesia. *The Journal of the Polynesian Society*, *103* (3), 255-298. Retrieved from http://www.jstor.org/stable/20706578

Kirch, P.V. (1994B). *The Wet and the Dry: Irrigation and Agricultural Intensification in Polynesia.* Chicago, IL: University of Chicago Press.

Kirch, P.V. (2004). Environmental and Ethnographic Background. In E. Conte & P.V. Kirch (Eds.), *Archaeological Investigations in the Mangareva Islands (Gambier Archipelago), French Polynesia* (pp 16-32). Berkeley, CA: Archaeological Research Facility, University of California: Berkeley.

Kirch, P.V. & Lepofsky, D. (1993). Polynesian Irrigation: Archaeological and Linguistic Evidence for Origins and Development. *Asian Perspectives*, *32* (2), 183-204. Retrieved from http://scholarspace.manoa.hawaii.edu/handle/10125/17030

Kuhlken, R. (2002). Intensive Agricultural Landscapes of Oceania. *Journal of Cultural Geography*, *19* (2), 161-195. DOI: 10.1080/08873630209478292

Lebo, S.A. (2004). Cultural History. In N.L. Evenhuis & L.G. Eldredge (Eds.), *Natural History of Nihoa and Necker Islands* (pp 32-44). Honolulu, HI: Bishop Museum Press.

Lepofsky, D. (1999). Gardens of Eden? An Ethnohistoric Reconstruction of Maohi (Tahitian) Cultivation. *Ethnohistory*, *46* (1), 1-29. Retrieved from http://www.jstor.org/stable/483427

Oliver, D.L. (1973). *Bougainville: A Personal History*. Melbourne, Australia: Melbourne University Press.

Peoples, J.G. (1991). Kosrae. In T.E. Hays (Ed.), *Encyclopaedia of World Cultures (Volume II: Oceania)* (pp 128-131). New York, NY: G.K. Hall & Co.

Roe, D. (1993*). Prehistory without Pots: Prehistoric Settlement and Economy of Northwest Guadalcanal, Solomon Islands*. Unpublished PhD thesis, Australian National University, Canberra. Retrieved from http://hdl.handle.net/1885/8040

Rolett, B.R. (2008). Avoiding Collapse: Pre-European Sustainability on Pacific Islands. *Quaternary International*, *184*, 4-10. DOI: 10.1016/j.quaint.2007.10.016.

Sand, C., Bole, J., & Ouetcho, A. (2007). What were the Real Numbers? The Question of Pre-Contact Population Densities in New Caledonia. In P.V. Kirch & J. Rallu (Eds.), *The Growth and Collapse of Pacific Island Societies.*(pp 306-325) Honolulu, HI: University of Hawaii Press.

Scheffler, H.W. (1965) *Choiseul Island Social Structure*. Berkeley, CA: University of California Press.

Smith, S.P. (1983). *Niue: The Island and its People*. Suva, Fiji: University of the South Pacific.

Speiser, F. (1990). *Ethnology of Vanuatu: an Early Twentieth Century Study.* Bathurst, Australia: Crawford House Press.

Spriggs, M. (1986). Landscape, Land Use and Political Transformation in Southern Melanesia. In in Kirch, P.V. (Ed.), *Island Societies: Archaeological Approaches to Evolution and Transformation* (pp 6-19). Cambridge, England: Cambridge University Press.

Spriggs, M. (1991). Preceded by Forest: Changing Interpretations of Landscape Change on Kaho'olawe, *Asian Perspectives*, *30* (1), 71-116.

Spriggs, M. (1997). *The Island Melanesians*. Oxford, England: Blackwell Publishers.

Spriggs, M. (2007). Population in a Vegetable Kingdom: Aneityum Island (Vanuatu) at European Contact in 1830. In P.V. Kirch & J. Rallu (Eds.), *The Growth and Collapse of Pacific Island Societies* (pp 278-305). Honolulu, HI: University of Hawai'i Press.

Stepien, E.R. (1988). *Ni'ihau: A Brief History.* Honolulu, HI: University of Hawaii at Manoa.

Walter, R. (1996). Settlement Pattern Archaeology in the Southern Cook Islands: A Review. *The Journal of the Polynesian Society*, *105* (1), 63-99. Retrieved from http://www.jstor.org/stable/20706646

Walter, R. & Anderson, A. (1995). Archaeology of Niue Island: Initial Results. *The Journal of the Polynesian Society*, *104* (4), 471-481. Retrieved from http://www.jstor.org.ezproxy.auckland.ac.nz/stable/20706637

Watters, R.F. (1958). Cultivation in Old Samoa. *Economic Geography*, 43 (4), 338-351. DOI: 10.2307/142351.

Weckler, J.E. (1943). *Polynesians: Explorers of the Pacific.* Washington, DC: Smithsonian Institution.

Weightman, B. (1989). *Agriculture in Vanuatu: a Historical Review*. Portsmouth, England: Grosvenor Press.

Yen, D.E. (1961). The Adaptation of Kumara by the New Zealand Maori. *The Journal of the Polynesian Society, 70* (3), 338-348. Retrieved from http://www.jstor.org.ezproxy.auckland.ac.nz/stable/20703913
