# Supplementary material for: Cultural and Environmental Predictors of Pre-European Deforestation on Pacific Islands
Source: PLoS One. 2016 May 27;11(5):e0156340. doi: 10.1371/journal.pone.0156340 (PMC4883741; doi:10.1371/journal.pone.0156340)
Supplement: S3 Table — (PDF) [file pone.0156340.s005.pdf]

S3 Table. Land Tenure coding scheme (adapted from Currie 2013 & Kushnick et al. 2014)			
**Code**	**Mode of intensification**	**Description**	**Example**
E	Elite ownership	Land ownership by elites typical	“At the time of contact landownership with the right of inheritance was recognized for those of the chiefly and commoner classes, with only the lower class, known as teuteu, being excluded” (Ferdon, 1996, p. 306).
I	Individual Ownership	Individual land owndership by common people typical (i.e. not just elites)	“Every Tannese boy receives a personal name that entitles him to several plots of land near a kava drinking ground” (Lindstrom 1996, p. 314).
O	Other ownership	Corporate (group or kin-based) land ownership or no ownership typical	"Local clan segments whose members know their exact genealogical relationship to each other are the largest corporate land-owning groups in the area, but sometimes smaller lineages emerge as de facto controllers of estates" (Lomas, 1979, p. 57).<br><br>"Garden land, which is only a temporary clearing in the forest, is not owned by any person or group" (Goodale, 1985, p. 230).
**Guidelines for land tenure:**<br><br>All forms of land ownership described as typical in a culture are coded as present. If two or more forms of land tenure are referred to within a given culture, these are all coded as present.			

References

Currie, T.E. (2013). Cultural evolution branches out: the phylogenetic approach in cross-cultural research. *Cross-Cultural Research*, 47, 102-130.

Ferdon, E. (1996) Tahiti. In D. Levinson & T. O'Leary (Eds.), *Encyclopedia of World Cultures* (Vol. 2: Oceania, pp. 305-307). NY: Macmillan.

Goodale, Jane (1985). Pig's teeth and skull cycles: both sides of the face of humanity. *American Ethnologist* 12(2): 228-244.

Kushnick G, Gray RD, & Jordan FM (2014) The sequential evolution of land tenure norms. *Evolution and Human Behavior* 35(4):309-318.

Lindstrom, L. (1996). Tanna. In D. Levinson & T. O'Leary (Eds.), *Encyclopedia of World Cultures* (Vol. 2: Oceania, pp. 313-315). New York: Macmillan Reference USA.

Lomas, P.W. (1979) Malanggans and manipulators: Land and politics in Northern New Ireland. *Oceania* 50(1): 53-66.
