# Supplementary material for: Cultural and Environmental Predictors of Pre-European Deforestation on Pacific Islands
Source: PLoS One. 2016 May 27;11(5):e0156340. doi: 10.1371/journal.pone.0156340 (PMC4883741; doi:10.1371/journal.pone.0156340)
Supplement: S4 Table — (PDF) [file pone.0156340.s006.pdf]

S4 Table. Land Tenure Norm coding

Datapoint	Island	Language (s)	Coding Justification	Code
Aneityum	Aneityum	Anejom	"Land is cultivated by the family but wasteland belongs to the tribe" (Humphreys, 1926, p. 109)	O
Austral Islands (Raivavae)	Raivavae	Rurutu	In her account of three islands in French Polynesia -- Raivavae, Rurutu, and Tubuai -- Lockwood (1994) states: "Land on the outer islands (unlike Tahiti) continues to be jointly owned by family groups" (Lockwood, 1994, p. 79).	O
Austral Islands (Rimatara)	Rimatara		In her account of three islands in French Polynesia -- Raivavae, Rurutu, and Tubuai -- Lockwood (1994) states: "Land on the outer islands (unlike Tahiti) continues to be jointly owned by family groups" (Lockwood, 1994, p. 79). This description is also taken to refer to the outer island Rimatara, although it is not explicitly mentioned.	O
Austral Islands (Rurutu)	Rurutu		In her account of three islands in French Polynesia -- Raivavae, Rurutu, and Tubuai -- Lockwood (1994) states: "Land on the outer islands (unlike Tahiti) continues to be jointly owned by family groups" (Lockwood, 1994, p. 79).	O
Austral Islands (Tubuai)	Tubuai		In her account of three islands in French Polynesia -- Raivavae, Rurutu, and Tubuai -- Lockwood (1994) states: "Land on the outer islands (unlike Tahiti) continues to be jointly owned by family groups" (Lockwood, 1994, p. 79).	O
Austral Islands (Rapa)	Rapa	Rapa	"Essential to the Rapan system of land tenure is the proposition that improvements (gardens, groves of trees, and houses) may be and usually are owned separately from the land on which they are located. Both territory and improvements are owned by ramages, known as opu." (Hanson, 1991, p. 274).	O
Bougainville	Bougainville	Banoni	Evidence indicates Austronesian	I

		Taiof	speakers on Bougainville had individual land ownership. Among the Haku: “Once a woman inherits land, she is the sole owner of it" (Regan & Griffin, 2005, p. 376).	
		Teop		
		Torau		
Choiseul	Choiseul	Babatana	“Ownership of land is by kin groups known as sinangge, but ownership of trees is by single persons. Because only flatter strips along the shoreline suitable for coconut plantations are really valuable and because such land is in very short supply, land-tenure disputes are common and difficult to settle” (Scheffler, 1996, p. 38)	O
		Ririo		
		Vaghua		
		Varisi		
Cook Islands (Aitutaki)	Aitutaki	Rarotongan	“When Rarotonga and Aitutaki are compared, interesting parallels emerge. Both were organized into chiefdoms, both had dispersed coastal settlement located along a major ecotone (the Maungaroa situation is excluded from this generalization), and on both islands land was divided into tapere with multiple ownership of the internal divisions.” (Bellwood, 1971, pp 156-157).	O
Cook Islands (Atiu)	Atiu		Crocombe & Crocombe (1991) include a discussion of modern landholding issues in addition to traditional ones, reading as follows: “Traditionally land was held for practical purposes by small, localized kin groups” (pp. 40-41).	O
Cook Islands (Mangaia)	Mangaia		“Mangaians view land tenure as a family issue. The land has never been surveyed, and people continue to reject this aspect of modern life. The management of land and sea resources rests with the Arongamana ("people of power"), a group of family-based chiefs that is a unique feature of Mangaia. The Arongamana determines who manages land and what one does with it. Even inheritance is not solely under the control of a family group but must be approved by the Arongamana. The Arongamana consists of representatives from every family on the island” (McCall, 2002, p. 198).	O

Cook Islands (Mau'ke)	Mau'ke		Crocombe & Crocombe (1991) include a discussion of modern landholding issues in addition to traditional ones, reading as follows: "Traditionally land was held for practical purposes by small, localized kin groups" (pp. 40-41).	O
Cook Islands (Mitiaro)	Mitiaro		Crocombe & Crocombe (1991) include a discussion of modern landholding issues in addition to traditional ones, reading as follows: "Traditionally land was held for practical purposes by small, localized kin groups" (pp. 40-41).	O
Cook Islands (Rarotonga)	Rarotonga		"When Rarotonga and Aitutaki are compared, interesting parallels emerge. Both were organized into chiefdoms, both had dispersed coastal settlement located along a major ecotone (the Maungaroa situation is excluded from this generalization), and on both islands land was divided into tapere with multiple ownership of the internal divisions." (Bellwood, 1971, pp 156-157).	O
Easter Island	Easter Island	Rapa Nui	"In traditional times, land was owned by lineages with dwelling and farm plots alloted to families. Since 1888 Chile has maintained ownership of all of Easter Island and has restricted the Easter Islanders to land in and around Hangoroa. Newlyweds are given a few acres of land for their use by the Chilean government" (Levinson & O'Leary, 1991, p. 54).	O
Efate	Efate	South Efate	The Nguna are also referred to as "Efate, Ngunese, Sesake" (Facey 1991: 242). Land tenure is described as follows: "Communal ownership of land is vested in matriclans. The pattern of actual land use, however, is a matter of individuals' pressing claims through diverse lines of connection. The strongest claim is through one's father's having worked the land previously. But claims made through one's mother or other relatives may also be made" (Facey 1991, p. 243). This is also consistent with Lane - "The limited evidence for the rest of the New Hebrides suggests that basic tenure	O

			patterns were similar everywhere, with connections to land transmitted unilineally through descent groups… " (Lane, 1971, p.254-5).	
Erromango (windward)	Erromango	Sye	"While the land belongs to the district group, gardens may be made by any man, in the bush, on that has been deserted by a former garden-maker, and these are the sole property of the maker as long as he continues to care for them…" (Humphreys, 1930, p.150).	O, I
Erromango (lee)		Ura	"While the land belongs to the district group, gardens may be made by any man, in the bush, on that has been deserted by a former garden-maker, and these are the sole property of the maker as long as he continues to care for them…" (Humphreys, 1930, p.150).	O, I
Espiritu Santo	Espiritu Santo	Araki (Southwest Santo)	"The limited evidence for the rest of the New Hebrides suggests that basic tenure patterns were similar everywhere, with connections to land transmitted unilineally through descent groups…On parts of Santo…the matrilineal base may be difficult to recognize and attitudes toward land may appear inexplicable" (Lane, 1971, pp. 254-5).	O
		Merei		
		Sakao (Port Olry)		
Fijian - Viti Levu (windward)	Viti Levu	Fijian (Bau)	"Land was held by the "family," which was defined more or less inclusively in different parts of Fiji. During the period of its rise to power, Bau struggled with Rewa for control of the delta and sought to impose tributary relationship on those they conquered. The colonial government defined principles of land tenure retrospectively, creating homogeneity in place of a system built of dynamism and change. They based their system at least in part on Bauan norms" (Routledge, 1996, p. 23).	O
Fijian - Viti Levu (lee)		Western Fijian (Navosa)	"Land was held by the "family," which was defined more or less inclusively in different parts of Fiji. During the period of its rise to power, Bau struggled with Rewa for control of the delta and sought to impose tributary relationship on those	O

			they conquered. The colonial government defined principles of land tenure retrospectively, creating homogeneity in place of a system built of dynamism and change. They based their system at least in part on Bauan norms” (Routledge, 1996, p. 23).	
Fijian - Lakeba	Lakeba	Fijian (Bau)	“Land was held by the "family," which was defined more or less inclusively in different parts of Fiji. During the period of its rise to power, Bau struggled with Rewa for control of the delta and sought to impose tributary relationship on those they conquered. The colonial government defined principles of land tenure retrospectively, creating homogeneity in place of a system built of dynamism and change. They based their system at least in part on Bauan norms” (Routledge, 1996, p. 23).	O
Fijian - Taveuni (Lee)	Taveuni (Lee)		“Land was held by the "family," which was defined more or less inclusively in different parts of Fiji. During the period of its rise to power, Bau struggled with Rewa for control of the delta and sought to impose tributary relationship on those they conquered. The colonial government defined principles of land tenure retrospectively, creating homogeneity in place of a system built of dynamism and change. They based their system at least in part on Bauan norms” (Routledge, 1996, p. 23).	O
Fijian - Taveuni (Wind)	Tavenui (Wind)		“Land was held by the "family," which was defined more or less inclusively in different parts of Fiji. During the period of its rise to power, Bau struggled with Rewa for control of the delta and sought to impose tributary relationship on those they conquered. The colonial government defined principles of land tenure retrospectively, creating homogeneity in place of a system built of dynamism and change. They based their system at least in part on Bauan norms” (Routledge, 1996, p. 23).	O
Fijian - Vanua Levu	Vanua Levu (Lee)		“Land was held by the "family," which was defined more or less inclusively in different parts of Fiji. During the period	O

(Lee)			of its rise to power, Bau struggled with Rewa for control of the delta and sought to impose tributary relationship on those they conquered. The colonial government defined principles of land tenure retrospectively, creating homogeneity in place of a system built of dynamism and change. They based their system at least in part on Bauan norms" (Routledge, 1996, p. 23).	
Fijian - Vanua Levu (Wind)	Vanua Levu (Wind)		"Land was held by the "family," which was defined more or less inclusively in different parts of Fiji. During the period of its rise to power, Bau struggled with Rewa for control of the delta and sought to impose tributary relationship on those they conquered. The colonial government defined principles of land tenure retrospectively, creating homogeneity in place of a system built of dynamism and change. They based their system at least in part on Bauan norms" (Routledge, 1996, p. 23).	O
Futuna and Alofi - Alofi	Alofi	Futuna, East	"The two halves of Futuna, Sigave and Alo, are distinct entities with separate land holdings; it is rare for a person to hold land in both kingdoms. Each saut or leader, is custodian of all lands in his territory, and in former times waged war in response to any violation of his lands. In each village the headman was responsible for ensuring that lands were properly used, but individual families could cultivate their household land and also use the vacant land behind the village. Some village land was maintained in production by a group of men in order to provide a bountiful supply of yams and kape for any large communal feast. Families depended on their household strip for day-to-day supplies of taro, bread-fruit, bananas, kape, and cassava. But in these days of large households, the men find it necessary to cultivate their own plantation land, and sometimes that of their wives, in order to grow enough to feed the family. Land rights are passed on to both sons and daughters, but a couple prefers to live on the man's land"	O, I

			(Pollock 1996, p. 66).	
Futuna and Alofi - Futuna	Futuna		“The two halves of Futuna, Sigave and Alo, are distinct entities with separate land holdings; it is rare for a person to hold land in both kingdoms. Each saut or leader, is custodian of all lands in his territory, and in former times waged war in response to any violation of his lands. In each village the headman was responsible for ensuring that lands were properly used, but individual families could cultivate their household land and also use the vacant land behind the village. Some village land was maintained in production by a group of men in order to provide a bountiful supply of yams and kape for any large communal feast. Families depended on their household strip for day-to-day supplies of taro, bread-fruit, bananas, kape, and cassava. But in these days of large households, the men find it necessary to cultivate their own plantation land, and sometimes that of their wives, in order to grow enough to feed the family. Land rights are passed on to both sons and daughters, but a couple prefers to live on the man's land” (Pollock, 1996, p. 66).	O, I
Guadalcanal (lee)	Guadalcanal	Ghari	"The chief importance of the clan organization is its application to land rights. The river deltas, where the recurring floods would destroy boundary marks, are treated as common property, but the rest of the land is cut into named blocks of varying acreage … These are grouped into series, each of which is bound up with a clan." (Hogbin, 1964, p 5); there are 5 matrilineal clans and >30,000 people.<br><br>“Use rights to land for adult males follows clan membership” (Gratton, 1991, p. 90).	O
Guadalcanal (windward)		Talise	"The chief importance of the clan organization is its application to land rights. The river deltas, where the recurring floods would destroy boundary marks, are treated as common property, but the rest of the	O

			land is cut into named blocks of varying acreage … These are grouped into series, each of which is bound up with a clan." (Hogbin, 1964, p 5); there are 5 matrilineal clans and >30,000 people.<br><br>“Use rights to land for adult males follows clan membership” (Gratton, 1991, p. 90)	
Hawaiian Islands (Hawaii (Lee))	Hawaii (Lee)	Hawaiian	“In the native Hawaiian conception land was not owned but "cared for." Use and access rights were allocated through the social hierarchy from the highest chiefs to their local land supervisors and thence to commoners. The most important administrative unit was a land section called the ahupua'a, which ideally ran from the mountain to the sea and contained a full range of productive zones. Typically a household had rights in a variety of microenvironments. The introduction of private land titles resulted in widespread dispossession in part because Hawaiians did not understand the implications of alienable property. The lands of the Kamehameha chiefly family descended to Princess Bernice Pauahi Bishop, whose estate supports the Kamehameha Schools in Honolulu for the education of Hawaiian children. The Hawaiian Home Lands, established by Congress in 1920, are leased to persons who can prove 50 percent Hawaiian ancestry. Originally conceived as a "back to the land" farming program, the Hawaiian Home Lands are now used primarily for house lots” (Linnekin, 1996, 96).	E
Hawaiian Islands (Hawaii (Wind))	Hawaii (Wind)		“In the native Hawaiian conception land was not owned but "cared for." Use and access rights were allocated through the social hierarchy from the highest chiefs to their local land supervisors and thence to commoners. The most important administrative unit was a land section called the ahupua'a, which ideally ran from the mountain to the sea and contained a full range of productive zones. Typically a household had rights in a variety of microenvironments. The introduction of private land titles resulted	E

			in widespread dispossession in part because Hawaiians did not understand the implications of alienable property. The lands of the Kamehameha chiefly family descended to Princess Bernice Pauahi Bishop, whose estate supports the Kamehameha Schools in Honolulu for the education of Hawaiian children. The Hawaiian Home Lands, established by Congress in 1920, are leased to persons who can prove 50 percent Hawaiian ancestry. Originally conceived as a "back to the land" farming program, the Hawaiian Home Lands are now used primarily for house lots” (Linnekin, 1996, 96).	
Hawaiian Islands (Kahoolawe)	Kahoolawe		“In the native Hawaiian conception land was not owned but "cared for." Use and access rights were allocated through the social hierarchy from the highest chiefs to their local land supervisors and thence to commoners. The most important administrative unit was a land section called the ahupua'a, which ideally ran from the mountain to the sea and contained a full range of productive zones. Typically a household had rights in a variety of microenvironments. The introduction of private land titles resulted in widespread dispossession in part because Hawaiians did not understand the implications of alienable property. The lands of the Kamehameha chiefly family descended to Princess Bernice Pauahi Bishop, whose estate supports the Kamehameha Schools in Honolulu for the education of Hawaiian children. The Hawaiian Home Lands, established by Congress in 1920, are leased to persons who can prove 50 percent Hawaiian ancestry. Originally conceived as a "back to the land" farming program, the Hawaiian Home Lands are now used primarily for house lots” (Linnekin, 1996, 96).	E
Hawaiian Islands (Kauai (Lee))	Kauai (Lee)		“In the native Hawaiian conception land was not owned but "cared for." Use and access rights were allocated through the social hierarchy from the highest chiefs to their local land supervisors and thence to commoners. The most	E

			important administrative unit was a land section called the ahupua'a, which ideally ran from the mountain to the sea and contained a full range of productive zones. Typically a household had rights in a variety of microenvironments. The introduction of private land titles resulted in widespread dispossession in part because Hawaiians did not understand the implications of alienable property. The lands of the Kamehameha chiefly family descended to Princess Bernice Pauahi Bishop, whose estate supports the Kamehameha Schools in Honolulu for the education of Hawaiian children. The Hawaiian Home Lands, established by Congress in 1920, are leased to persons who can prove 50 percent Hawaiian ancestry. Originally conceived as a "back to the land" farming program, the Hawaiian Home Lands are now used primarily for house lots” (Linnekin, 1996, 96).	
Hawaiian Islands (Kauai (Wind))	Kauai (Wind)		“In the native Hawaiian conception land was not owned but "cared for." Use and access rights were allocated through the social hierarchy from the highest chiefs to their local land supervisors and thence to commoners. The most important administrative unit was a land section called the ahupua'a, which ideally ran from the mountain to the sea and contained a full range of productive zones. Typically a household had rights in a variety of microenvironments. The introduction of private land titles resulted in widespread dispossession in part because Hawaiians did not understand the implications of alienable property. The lands of the Kamehameha chiefly family descended to Princess Bernice Pauahi Bishop, whose estate supports the Kamehameha Schools in Honolulu for the education of Hawaiian children. The Hawaiian Home Lands, established by Congress in 1920, are leased to persons who can prove 50 percent Hawaiian ancestry. Originally conceived as a "back to the land" farming program, the Hawaiian Home Lands are now used primarily for house lots” (Linnekin,	E

			1996, 96).	
Hawaiian Islands (Lanai)	Lanai		“In the native Hawaiian conception land was not owned but "cared for." Use and access rights were allocated through the social hierarchy from the highest chiefs to their local land supervisors and thence to commoners. The most important administrative unit was a land section called the ahupua'a, which ideally ran from the mountain to the sea and contained a full range of productive zones. Typically a household had rights in a variety of microenvironments. The introduction of private land titles resulted in widespread dispossession in part because Hawaiians did not understand the implications of alienable property. The lands of the Kamehameha chiefly family descended to Princess Bernice Pauahi Bishop, whose estate supports the Kamehameha Schools in Honolulu for the education of Hawaiian children. The Hawaiian Home Lands, established by Congress in 1920, are leased to persons who can prove 50 percent Hawaiian ancestry. Originally conceived as a "back to the land" farming program, the Hawaiian Home Lands are now used primarily for house lots” (Linnekin, 1996: 96).	E
Hawaiian Islands (Maui (Lee))	Maui (Lee)		“In the native Hawaiian conception land was not owned but "cared for." Use and access rights were allocated through the social hierarchy from the highest chiefs to their local land supervisors and thence to commoners. The most important administrative unit was a land section called the ahupua'a, which ideally ran from the mountain to the sea and contained a full range of productive zones. Typically a household had rights in a variety of microenvironments. The introduction of private land titles resulted in widespread dispossession in part because Hawaiians did not understand the implications of alienable property. The lands of the Kamehameha chiefly family descended to Princess Bernice Pauahi Bishop, whose estate supports the Kamehameha Schools in Honolulu for the education of Hawaiian children.	E

			The Hawaiian Home Lands, established by Congress in 1920, are leased to persons who can prove 50 percent Hawaiian ancestry. Originally conceived as a "back to the land" farming program, the Hawaiian Home Lands are now used primarily for house lots" (Linnekin, 1996, 96).	
Hawaiian Islands (Maui (Wind))	Maui (Wind)		"In the native Hawaiian conception land was not owned but "cared for." Use and access rights were allocated through the social hierarchy from the highest chiefs to their local land supervisors and thence to commoners. The most important administrative unit was a land section called the ahupua'a, which ideally ran from the mountain to the sea and contained a full range of productive zones. Typically a household had rights in a variety of microenvironments. The introduction of private land titles resulted in widespread dispossession in part because Hawaiians did not understand the implications of alienable property. The lands of the Kamehameha chiefly family descended to Princess Bernice Pauahi Bishop, whose estate supports the Kamehameha Schools in Honolulu for the education of Hawaiian children. The Hawaiian Home Lands, established by Congress in 1920, are leased to persons who can prove 50 percent Hawaiian ancestry. Originally conceived as a "back to the land" farming program, the Hawaiian Home Lands are now used primarily for house lots" (Linnekin, 1996, 96).	E
Hawaiian Islands (Molokai (Lee))	Molokai (Lee)		"In the native Hawaiian conception land was not owned but "cared for." Use and access rights were allocated through the social hierarchy from the highest chiefs to their local land supervisors and thence to commoners. The most important administrative unit was a land section called the ahupua'a, which ideally ran from the mountain to the sea and contained a full range of productive zones. Typically a household had rights in a variety of microenvironments. The introduction of private land titles resulted in widespread dispossession in part	E

			because Hawaiians did not understand the implications of alienable property. The lands of the Kamehameha chiefly family descended to Princess Bernice Pauahi Bishop, whose estate supports the Kamehameha Schools in Honolulu for the education of Hawaiian children. The Hawaiian Home Lands, established by Congress in 1920, are leased to persons who can prove 50 percent Hawaiian ancestry. Originally conceived as a "back to the land" farming program, the Hawaiian Home Lands are now used primarily for house lots" (Linnekin, 1996, 96).	
Hawaiian Islands (Molokai (Wind))	Molokai<br><br>(Wind)		"In the native Hawaiian conception land was not owned but "cared for." Use and access rights were allocated through the social hierarchy from the highest chiefs to their local land supervisors and thence to commoners. The most important administrative unit was a land section called the ahupua'a, which ideally ran from the mountain to the sea and contained a full range of productive zones. Typically a household had rights in a variety of microenvironments. The introduction of private land titles resulted in widespread dispossession in part because Hawaiians did not understand the implications of alienable property. The lands of the Kamehameha chiefly family descended to Princess Bernice Pauahi Bishop, whose estate supports the Kamehameha Schools in Honolulu for the education of Hawaiian children. The Hawaiian Home Lands, established by Congress in 1920, are leased to persons who can prove 50 percent Hawaiian ancestry. Originally conceived as a "back to the land" farming program, the Hawaiian Home Lands are now used primarily for house lots" (Linnekin, 1996, 96).	E
Hawaiian Islands (Necker)	Necker		"In the native Hawaiian conception land was not owned but "cared for." Use and access rights were allocated through the social hierarchy from the highest chiefs to their local land supervisors and thence to commoners. The most important administrative unit was a land	E

			section called the ahupua'a, which ideally ran from the mountain to the sea and contained a full range of productive zones. Typically a household had rights in a variety of microenvironments. The introduction of private land titles resulted in widespread dispossession in part because Hawaiians did not understand the implications of alienable property. The lands of the Kamehameha chiefly family descended to Princess Bernice Pauahi Bishop, whose estate supports the Kamehameha Schools in Honolulu for the education of Hawaiian children. The Hawaiian Home Lands, established by Congress in 1920, are leased to persons who can prove 50 percent Hawaiian ancestry. Originally conceived as a "back to the land" farming program, the Hawaiian Home Lands are now used primarily for house lots” (Linnekin, 1996, 96).	
Hawaiian Islands (Nihoa)	Nihoa		“In the native Hawaiian conception land was not owned but "cared for." Use and access rights were allocated through the social hierarchy from the highest chiefs to their local land supervisors and thence to commoners. The most important administrative unit was a land section called the ahupua'a, which ideally ran from the mountain to the sea and contained a full range of productive zones. Typically a household had rights in a variety of microenvironments. The introduction of private land titles resulted in widespread dispossession in part because Hawaiians did not understand the implications of alienable property. The lands of the Kamehameha chiefly family descended to Princess Bernice Pauahi Bishop, whose estate supports the Kamehameha Schools in Honolulu for the education of Hawaiian children. The Hawaiian Home Lands, established by Congress in 1920, are leased to persons who can prove 50 percent Hawaiian ancestry. Originally conceived as a "back to the land" farming program, the Hawaiian Home Lands are now used primarily for house lots” (Linnekin, 1996, 96).	E

Hawaiian Islands (Niihau)	Niihau		“In the native Hawaiian conception land was not owned but "cared for." Use and access rights were allocated through the social hierarchy from the highest chiefs to their local land supervisors and thence to commoners. The most important administrative unit was a land section called the ahupua'a, which ideally ran from the mountain to the sea and contained a full range of productive zones. Typically a household had rights in a variety of microenvironments. The introduction of private land titles resulted in widespread dispossession in part because Hawaiians did not understand the implications of alienable property. The lands of the Kamehameha chiefly family descended to Princess Bernice Pauahi Bishop, whose estate supports the Kamehameha Schools in Honolulu for the education of Hawaiian children. The Hawaiian Home Lands, established by Congress in 1920, are leased to persons who can prove 50 percent Hawaiian ancestry. Originally conceived as a "back to the land" farming program, the Hawaiian Home Lands are now used primarily for house lots” (Linnekin, 1996, 96).	E
Hawaiian Islands (Oahu ((Lee))	Oahu (Lee)		“In the native Hawaiian conception land was not owned but "cared for." Use and access rights were allocated through the social hierarchy from the highest chiefs to their local land supervisors and thence to commoners. The most important administrative unit was a land section called the ahupua'a, which ideally ran from the mountain to the sea and contained a full range of productive zones. Typically a household had rights in a variety of microenvironments. The introduction of private land titles resulted in widespread dispossession in part because Hawaiians did not understand the implications of alienable property. The lands of the Kamehameha chiefly family descended to Princess Bernice Pauahi Bishop, whose estate supports the Kamehameha Schools in Honolulu for the education of Hawaiian children. The Hawaiian Home Lands, established	E

			by Congress in 1920, are leased to persons who can prove 50 percent Hawaiian ancestry. Originally conceived as a "back to the land" farming program, the Hawaiian Home Lands are now used primarily for house lots” (Linnekin, 1996, 96).	
Hawaiian Islands<br><br>(Oahu (Wind))	Oahu (Wind)		“In the native Hawaiian conception land was not owned but "cared for." Use and access rights were allocated through the social hierarchy from the highest chiefs to their local land supervisors and thence to commoners. The most important administrative unit was a land section called the ahupua'a, which ideally ran from the mountain to the sea and contained a full range of productive zones. Typically a household had rights in a variety of microenvironments. The introduction of private land titles resulted in widespread dispossession in part because Hawaiians did not understand the implications of alienable property. The lands of the Kamehameha chiefly family descended to Princess Bernice Pauahi Bishop, whose estate supports the Kamehameha Schools in Honolulu for the education of Hawaiian children. The Hawaiian Home Lands, established by Congress in 1920, are leased to persons who can prove 50 percent Hawaiian ancestry. Originally conceived as a "back to the land" farming program, the Hawaiian Home Lands are now used primarily for house lots” (Linnekin, 1996, 96).	E
Henderson Island	Henderson	NA	Uninhabited	NA
Kosrae	Kosrae	Kusaie	“Before the middle decades of the nineteenth century, the principal chief controlled the allocation of all the land on the island. He allocated control over particular districts, with their natural resources and commoner residents, to other members of the noble class. Commoners, and in theory other members of the nobility, used the land only by his leave. In return, commoners were obliged to supply regular tribute and labor services to the chief to whom	E

			their district was assigned. Today ownership is in the hands of individuals, although a group of siblings will occasionally maintain Control over plots” (Peoples, 1996, 129).	
Malaita	Malaita	Lau	Keesing (1991) lists Lau, Sa'a, and Areare as ethnonyms for Malaita, and describes land tenure as follows: "Primary rights to land are obtained through tracing patrifiliation, but secondary rights are also granted to those with maternal links to ancestors” (p. 161).	O
		Lau (North)		
		Lau (Walade)		
		Saa		
		Areare (Waiahaa Village)		
		Areare (Maasupa Village)		
Mangareva (Lee)	Mangareva (Lee)	Mangareva	"Every part of the land had an owner…the land was owned by the aristocracy…the landowner usually leased land to farmers…" (Buck 1938, pp. 161-2).	E
Mangareva (Wind)	Mangareva (Wind)		"Every part of the land had an owner…the land was owned by the aristocracy…the landowner usually leased land to farmers…" (Buck 1938, pp. 161-2).	E
Marquesas (Eiao)	Eiao	Marquesan	Thomas (1991) describes the culture and social organization of the Marquesas Islands, including "Nukuhiva, Ua Pou, Ua Huka, Fatuiva, Tahuata, and Hova Oa" and Eiao (p. 188). In the section on Land Tenure (p. 189) it states: "In the early nineteenth century there was great emphasis on the rights of firstborn children to inherit property."	I
Marquesas (Fatu Hiva)	Fatu Hiva		Thomas (1991) describes the culture and social organization of the Marquesas Islands, including "Nukuhiva, Ua Pou, Ua Huka, Fatuiva, Tahuata, and Hova Oa" and Eiao (p. 188). In the section on Land Tenure (p. 189) it	I

			states: "In the early nineteenth century there was great emphasis on the rights of firstborn children to inherit property."	
Marquesas (Hatuta'a)	Hatuta'a		Thomas (1991) describes the culture and social organization of the Marquesas Islands, including "Nukuhiva, Ua Pou, Ua Huka, Fatuiva, Tahuata, and Hova Oa" and Eiao (p. 188). In the section on Land Tenure (p. 189) it states: "In the early nineteenth century there was great emphasis on the rights of firstborn children to inherit property."	I
Marquesas (Hiva Oa)	Hiva Oa		Thomas (1991) describes the culture and social organization of the Marquesas Islands, including "Nukuhiva, Ua Pou, Ua Huka, Fatuiva, Tahuata, and Hova Oa" and Eiao (p. 188). In the section on Land Tenure (p. 189) it states: "In the early nineteenth century there was great emphasis on the rights of firstborn children to inherit property."	I
Marquesas (Nukuhiva)	Nukuhiva		Thomas (1991) describes the culture and social organization of the Marquesas Islands, including "Nukuhiva, Ua Pou, Ua Huka, Fatuiva, Tahuata, and Hova Oa" and Eiao (p. 188). In the section on Land Tenure (p. 189) it states: "In the early nineteenth century there was great emphasis on the rights of firstborn children to inherit property."	I
Marquesas (Tahuata)	Tahuata		Thomas (1991) describes the culture and social organization of the Marquesas Islands, including "Nukuhiva, Ua Pou, Ua Huka, Fatuiva, Tahuata, and Hova Oa" and Eiao (p. 188). In the section on Land Tenure (p. 189) it states: "In the early nineteenth century there was great emphasis on the rights of firstborn children to inherit property."	I
Marquesas (Ua Huka)	Ua Huka		Thomas (1991) describes the culture and social organization of the Marquesas Islands, including "Nukuhiva, Ua Pou, Ua Huka, Fatuiva, Tahuata, and Hova Oa" and Eiao (p. 188). In the section on Land Tenure (p. 189) it states: "In the early nineteenth century there was great emphasis on the rights	I

			of firstborn children to inherit property."	
Marquesas (Ua Pou)	Ua Pou		Thomas (1991) describes the culture and social organization of the Marquesas Islands, including "Nukuhiva, Ua Pou, Ua Huka, Fatuiva, Tahuata, and Hova Oa" and Eiao (p. 188). In the section on Land Tenure (p. 189) it states: "In the early nineteenth century there was great emphasis on the rights of firstborn children to inherit property."	I
New Britain (lee)	New Britain	Kuanua<br>Lunga Lunga (Minigir)<br>Maututu<br>Amara<br>Nakanai (Bileki Dialect)	Kuanua:<br><br>Tolai "is also known as 'Kuanua'" (p.333); "In theory, all land is 'owned'--that is, is is vested in the group called a vunatarai, a matrilineal clan whose members may be dispersed through many villages" (Epstein 1991, p.334).<br><br>Nakanai:<br><br>"Land is vested in the clan, and use rights to garden on it are granted by senior resident male to non-clan memmbers such as children and grandchildren of men of the clan and phratry mates. With the expanding population and much land permanenty under cash crops, clan segments have begun to be less generous to outsiders. Trees are inherited separately but revert to the landowners if no direct descendents of teh planter remain in the area. Some productive reefs are also claimed by clans" (Chowning, 1991a, p. 141).	O
New Britain (Windward)		Kaulong (Au Village)<br>Sengseng	Kaulong:<br><br>"Garden land, which is only a temporary clearing in the forest, is not owned by any person or group" (Goodale, 1985, p.230).<br><br>Sengseng:<br><br>“Surprisingly for horticulturalists, gardening land is not owned, though the site of a men's house and the trees	O

			planted nearby are. It is believed that taro grows best near where an ancestor is buried, but any descendant of a Person who once lived in a settlement can make a garden in the vicinity. There is no shortage of land" (Chowning, 1991b, p. 298).	
New Caledonia	New Caledonia	Canala<br>Jawe<br>Nelemwa	Saussol (1971) speaks of customary land tenure in New Caledonia as such: "the land belonged to the first established clan that worked it, whose origins were so distant that it was often to have sprung from the very soil" (p. 242); what is meant by a "clan" is ucnlear, but that a chief surrendered land to the colonial powers in 1895 among the Canala suggests that this is group ownership.	O
New Ireland	New Ireland	Tigak<br>Kara West,<br>Nalik<br>Patpatar<br>Kandas	Tigak:<br><br>"Local clan segments whose members know their exact genealogical relationship to each other are the largest corporate land-owning groups in the area, but sometimes smaller lineages emerge as de facto controllers of estates" (Lomas, 1979, p. 57).	O
New Zealand (North Island)	North Island	Maori	"Nearly all land was owned by the various descent groups or tribes. Each group controlled a parcel of Tribal territory and granted rights of usufruct and occupation to its members. Only the group could alienate the descent group's land, and then only with the permission of the entire tribe. Border disputes were a common source of fighting. The nuclear family (whanau) of a descent group held rights to specific resources and parcels of land, which could be conveyed to the members' children. Rights of use could be extended to nonmembers only with the permission of the entire descent group"	O

			(Latham, 1996, p. 177).	
New Zealand (South Island (East Coast))	South Island (East Coast)	South Island Maori	“Nearly all land was owned by the various descent groups or tribes. Each group controlled a parcel of Tribal territory and granted rights of usufruct and occupation to its members. Only the group could alienate the descent group's land, and then only with the permission of the entire tribe. Border disputes were a common source of fighting. The nuclear family (whanau) of a descent group held rights to specific resources and parcels of land, which could be conveyed to the members' children. Rights of use could be extended to nonmembers only with the permission of the entire descent group” (Latham, 1996, p. 177).	O
New Zealand (South Island (West Coast))	South Island (West Coast)		“Nearly all land was owned by the various descent groups or tribes. Each group controlled a parcel of Tribal territory and granted rights of usufruct and occupation to its members. Only the group could alienate the descent group's land, and then only with the permission of the entire tribe. Border disputes were a common source of fighting. The nuclear family (whanau) of a descent group held rights to specific resources and parcels of land, which could be conveyed to the members' children. Rights of use could be extended to nonmembers only with the permission of the entire descent group” (Latham, 1996, p. 177).	O
Niue	Niue	Niue	“In the mid-1970s only 1 percent of the island was in Crown hands and 4 percent was held in the form of lease in perpetuity. Of the remainder, only 5 percent had been formally surveyed and registered, while 90 percent was listed as under "customary tenure." Much of the coastline, a few areas of forest, and church greens are under village control, but the bulk of the island is divided between specific family groups, or magafaoa. Every such piece of land, or fonua, has an ancestral "source," most often a male who lived several generations before the oldest living	O

			persons now associated with it, and each household controls and utilizes a number of fonua. Inheritance rights are granted to adopted children, and outsiders sometimes are given short-term use rights. Family members living away never totally abandon their rights" (Ryan, 2002, p. 229).	
Pitcairn	Pitcairn	NA	Uninhabited when discovered by Europeans, though was likely inhabited by people from Mangareva.	NA
Rotuma	Rotuma	Rotuman	"Land is important to Rotumans for its symbolic significance as well as for its subsistence value. The main landholding unit is the kainaga, a bilateral group based upon common descent from ancestors who resided at, and held rights in, a named house site (fuaq ri). Each person is considered to have rights in the fuaq ri of his eight greatgrandparents, although typically rights are exercised selectively. Associated with each fuaq ri are sections of bush land, and membership in a given kainaga entitles one to rights in this land. The person who lives on the fuaq ri acts as steward of the land and controls access. He, or she, is obligated to grant usufructuary rights to kainaga members for any reasonable request. At times land has been sold or given for services to specific individuals, but over generations it becomes kainaga land again. When the population of the island approached its highest levels, during the 1950s and 1960s, land disputes intensified and access was generally restricted to close relatives. In recent years, however, out-migration has relieved tensions and the main problem now is often to determine which of a set of siblings will remain behind to steward the land and care for aging parents" (Howard, 1996, pp. 281-2).	O
Samoa (Savaii)	Savaii	Samoan	"Aboriginally, the widest social unit for landownership was the community (nu'u). Its domain included all the territory from the central mountain ridge to the reef. The heads (matai) of the different descent groups ('āiga) of the	O
Samoa (Upolu)	Upolu			

			community were entitled to claim blocks of land for themselves and their dependents. Overall authority over lands, however, was vested in the council of matai (fono), whose members could revoke ownership of the respective 'āiga. Individuals had the right to occupy and cultivate the land of the descent group to which they belonged. When Western Samoa became independent, 80.5 percent of its territory was still considered customary land, administered outside the statute law in accordance with traditional principles of tenure; 3.7 percent of the land was freehold; 11.3 percent was government land; and the Western Samoan Trust Estate Corporation owned 4.5 percent. American Samoa, too, has provisions that restrict ownership of land to Samoans" (Bargatzky, 1996, p. 287).	
San Cristobal	San Cristobal	Arosi	Levinson & O'Leary (1991) includes a short description San Cristobal peoples—the Arosi, Bauro, Kahua, and Tawarafa—including the statement about land tenure: "Land is owned by the resident extended family" (p. 289).	O
		Fagani		
		Bauro (Haununu)		
		Kahua		
		Bauro (Pawa Village)		
		Bauro (Baroo Village)		
Santa Isabel	Santa Isabel	Laghu (Samasodu)	Cheke Holo:<br><br>" In societies as yet unaffected by the advent of church and government, ties of kinship, formed particularly by descent from matrilineal ancestors and attachments to ancestral lands, constituted the basic parameters of social life. Lineages of people who could	O
		Zabana (Kia)		

		Kokota	trace descent from a common ancestress composed the primary landholding groups, as they do today" (White, 1991, p. 33).	
		Blablanga		
		Cheke Holo	"Athough my interpretations pertain primarily to one of the island's four major language groups (Cheke Holo), there are enough similarities across the island to refer to 'Santa Isabel chiefs'" (White, 1997, p. 234).<br><br>Zabana Kia:<br><br>"Each and every member was regarded as holding an equal share in the group's land and had the right to be heard in discussions regarding any projected disposal of the group rights in any or all of the land which was owned" (Allan, 1988, p. 14).	
Society Islands (Bora Bora)	Bora Bora	Tahitian (Modern)	"At the time of contact landownership with the right of inheritance was recognized for those of the chiefly and commoner classes, with only the lower class, known as teuteu, being excluded. Such lands were subject to taxation in kind by the ruling chiefs who could banish an owner if such taxes were not forthcoming. Missionary activity in the nineteenth century seems to have resulted in at least some of the teuteu class obtaining land rights" (Ferdon, 1996, p. 306).	E
Society Islands (Huahine)	Huahine		"At the time of contact landownership with the right of inheritance was recognized for those of the chiefly and commoner classes, with only the lower class, known as teuteu, being excluded. Such lands were subject to taxation in kind by the ruling chiefs who could banish an owner if such taxes were not forthcoming. Missionary activity in the nineteenth century seems to have resulted in at least some of the teuteu class obtaining land rights" (Ferdon, 1996, p. 306).	E

Society Islands (Maupiti)	Maupiti		“At the time of contact landownership with the right of inheritance was recognized for those of the chiefly and commoner classes, with only the lower class, known as teuteu, being excluded. Such lands were subject to taxation in kind by the ruling chiefs who could banish an owner if such taxes were not forthcoming. Missionary activity in the nineteenth century seems to have resulted in at least some of the teuteu class obtaining land rights” (Ferdon, 1996, p. 306).	E
Society Islands (Moorea)	Moorea		“At the time of contact landownership with the right of inheritance was recognized for those of the chiefly and commoner classes, with only the lower class, known as teuteu, being excluded. Such lands were subject to taxation in kind by the ruling chiefs who could banish an owner if such taxes were not forthcoming. Missionary activity in the nineteenth century seems to have resulted in at least some of the teuteu class obtaining land rights” (Ferdon, 1996, p. 306).	E
Society Islands (Raiatea)	Raiatea		“At the time of contact landownership with the right of inheritance was recognized for those of the chiefly and commoner classes, with only the lower class, known as teuteu, being excluded. Such lands were subject to taxation in kind by the ruling chiefs who could banish an owner if such taxes were not forthcoming. Missionary activity in the nineteenth century seems to have resulted in at least some of the teuteu class obtaining land rights” (Ferdon, 1996, p. 306).	E
Society Islands (Tahaa)	Tahaa		“At the time of contact landownership with the right of inheritance was recognized for those of the chiefly and commoner classes, with only the lower class, known as teuteu, being excluded. Such lands were subject to taxation in kind by the ruling chiefs who could banish an owner if such taxes were not forthcoming. Missionary activity in the nineteenth century seems to have resulted in at least some of the teuteu	E

			class obtaining land rights” (Ferdon, 1996, p. 306).	
Society Islands (Tahiti)	Tahiti		“At the time of contact landownership with the right of inheritance was recognized for those of the chiefly and commoner classes, with only the lower class, known as teuteu, being excluded. Such lands were subject to taxation in kind by the ruling chiefs who could banish an owner if such taxes were not forthcoming. Missionary activity in the nineteenth century seems to have resulted in at least some of the teuteu class obtaining land rights” (Ferdon, 1996, p. 306).	E
Tanna	Tanna	Kwamera<br>Lenakel<br>Tanna (Southwest)	“Every Tannese boy receives a personal name that entitles him to several plots of land near a kava drinking ground. Women's names have no land entitlements. A name also may entitle a male bearer to perform various ritual acts, to control a section of traditional road, and so on. Every family possesses a limited number of names that are used each generation. If a man has no sons, he adopts boys (or other grown men) by giving them one of his names. In actual practice, the exact connection between a particular personal name and its associated lands is often disputed. Garden land, however, is plentiful, except in a few locales. Moreover, most people neither live nor garden upon their own lands; permission to use another's land is usually readily obtained” (Lindstrom 1996, p. 314).	I
Tikopia	Tikopia	Tikopia	“All the land of Tikopia is divided into orchards (tofi) of palms and fruit trees and into open gardens (vao), marked off into plots for annual cropping. Every orchard and garden plot is owned as of ancestral right by a distinct lineage group, with titular supreme rights exercised by the clan chief. (A similar system operates in overseas Tikopia settlements that have agricultural lands.) Within the lineage land, rights to produce are held by individual cultivators. By ancient custom, vacant garden land may be used for a season	O

			by other than its owners, on payment of a proportion of the crop. Permanent transfers of land from one group to another were rare, but historically transfers sometimes occurred when a chief gave some land to a daughter on her marriage. Sale of land is unknown. No land on Tikopia is held by other than Tikopia people” (Firth, 1996, p. 325).	
Tonga (Tongatapu)	Tongatapu	Tongan	“Current Tongan law guarantees that every male over the age of 16 should receive an allotment of land: an 'api of 3.3 hectares for agricultural purposes and 0.16 of a hectare as a site for a home. Because of population growth and limited natural resources, however, thousands of Tongan males are landless today. Prior to the Tongan constitution, established in 1875 by King George Tupou I (1797-1893), land rights in Tonga were vested with an extended kinship group, the ha'a, a corporate landholding and propertysharing descent group. The leadership of the ha'a distributed resources to members. In 1875, however, all land was acquired by the Crown for redistribution to a newly created class of hereditary nobles (nopele) for eventual redistribution to the people” (Urbanowicz, 1996, p. 337).	O
Makatea	Makatea	Tuamotu	Tuamotu is an ethnonym for Raroia: "Atoll land was divided into districts with the land owned by a combination of lineally and laterally extended kin groups...Families are now smaller and nuclear in form, with an emphasis on individual ownership of property" (Levinson & O’Leary, 1991, pp. 276-277).	O
Yap	Yap	Yapese	“Rights to land, lagoon, other fishing and agricultural resources, and village authority are held corporately by the patrilineal estate group. The heeeads of estates in consultation with their junior members excercise authority over the rights on behalf of members. Male members have use rights to estate resources with which they may support a wife and children. Succession to headship is based on generation and	O

			seniority" (Lingenfelter, 1991, p. 392).	

References

Allan, C.H. (1988). Land law and custom in Ysabel: Postwar change and development. *The Journal of the Polynesian Society* 97(1): 7-30.

Bargatzky, T. (1996). Samoa. In D. Levinson & T. O'Leary (Eds.), *Encyclopedia of World Cultures* (Vol. 2: Oceania, pp. 286-289). New York: Macmillan Reference USA.

Bellwood, P.S. (1971). Varieties of Ecological Adaptation in the Southern Cook Islands. *Archaeology and Physical Anthropology in Oceania*, *6* (2), 145-169. Retrieved from http://www.jstor.org/stable/40386143

Buck, P. (1938) Ethnology of Mangareva. *Bernice P. Bishop Museum Bulletin* 157.

Chowning, A. (1991a) Lakalai. In D. Levinson & T. O'Leary (Eds.), *Encyclopedia of World Cultures* (Vol. 2: Oceania, pp. 139-143). NY: Macmillan.

Chowning, A. (1991b) Sengseng. In D. Levinson & T. O'Leary (Eds.), *Encyclopedia of World Cultures* (Vol. 2: Oceania, pp. 295-298). NY: Macmillan.

Crocombe, R, and Crocombe, M.T.. (1991) Cook Islanders. In D. Levinson & T. O'Leary (Eds.), *Encyclopedia of World Cultures* (Vol. 2: Oceania, pp. 40-42). NY: Macmillan.

Epstein, A.L. (1991). Tolai. In T.E. Hays, (Ed.), *Encyclopaedia of World Cultures (Volume II: Oceania)* (pp 333-336). New York, NY: G.K. Hall & Co.

Facey, E. (1991). Nguna. In *Encyclopedia of World Cultures* (Vol. 2: Oceania, pp. #-#). New York: Macmillan Reference USA.

Ferdon, E. (1996) Tahiti. In D. Levinson & T. O'Leary (Eds.), *Encyclopedia of World Cultures* (Vol. 2: Oceania, pp. 305-307). NY: Macmillan.

Firth, R. (1996). Tikopia. In D. Levinson & T. O'Leary (Eds.), *Encyclopedia of World Cultures* (Vol. 2: Oceania, pp. 324-327). New York: Macmillan Reference USA.

Goodale, Jane (1985). Pig's teeth and skull cycles: both sides of the face of humanity. *American Ethnologist* 12(2): 228-244.

Gratton, N.E. (1991). Guadalcanal. In T.E. Hays (Ed.*), Encyclopaedia of World Cultures (Volume II: Oceania)* (pp. 88-92). New York, NY: G.K. Hall & Co.

Hanson, F.A. (1991) Rapa. In D. Levinson & T. O'Leary (Eds.), *Encyclopedia of World Cultures* (Vol. 2: Oceania, pp. 273-276). NY: Macmillan.

Hogbin, Ian (1964). *A Guadalcanal society: The Kaoka speakers*. NY: Holt, Rhinehart and Wilson.

Howard, A. (1996). Rotuma. In D. Levinson & T. O'Leary (Eds.), *Encyclopedia of World Cultures* (Vol. 2: Oceania, pp. 280-283). New York: Macmillan Reference USA.

Humphreys, C. B. (1926) *The southern New Hebrides: an ethnological record*. Cambridge University Press.

Keesing, R. (1991). Malaita. In D. Levinson & T. O'Leary (Eds.), *Encyclopedia of World Cultures* (Vol. 2: Oceania, pp. 160-163). New York: Macmillan Reference USA.

Lane R, 1971. The New Hebrides: Land tenure without land policy. In: R Crocombe (ed.) *Land tenure in the Pacific (*pp. 248-271). Melbourne: Oxford University Press.

Latham, C. (1996). Maori. In D. Levinson & T. O'Leary (Eds.), *Encyclopedia of World Cultures* (Vol. 2: Oceania, pp. 176-179). New York: Macmillan Reference USA

Levinson, D., & O'Leary, T. (1991). San Cristobal. In *Encyclopedia of World Cultures* (Vol. 2: Oceania, pp. #-#). New York: Macmillan Reference USA.

Levinson, D., & O'Leary, T. (1991). *Encyclopedia of World Cultures* (Vol. 2: Oceania). New York: Macmillan Reference USA.

Lindstrom, L. (1996). Tanna. In D. Levinson & T. O'Leary (Eds.), *Encyclopedia of World Cultures* (Vol. 2: Oceania, pp. 313-315). New York: Macmillan Reference USA.

Lingenfelter, SG (1991) Yap. In *Encyclopedia of World Cultures* (Vol. 2: Oceania, pp. 391-394). NY: Macmillan Reference USA.

Linnekin, J. (1996). Hawaiians. In D. Levinson & T. O'Leary (Eds.), *Encyclopedia of World Cultures* (Vol. 2: Oceania, pp. 95-97). New York: Macmillan Reference USA.

Lockwood, V. (1994) The impact of development on rural Tahitian women: a comparison of three islands. *Urban Anthropology and Studies of Cultural Systems and World Economic Development* 23(1): 73-96.

Lomas, P.W. (1979) Malanggans and manipulators: Land and politics in Northern New Ireland. *Oceania* 50(1): 53-66.

McCall, G. (2002). Mangaians. In *Encyclopedia of World Cultures* (Vol. Supplement, pp. 197-200). New York: Macmillan Reference USA.

Peoples, J. (1996). Kosrae. In D. Levinson & T. O'Leary (Eds.), *Encyclopedia of World Cultures* (Vol. 2: Oceania, pp. 128-131). New York: Macmillan Reference USA.

Pollock, N. (1996). Futuna. In D. Levinson & T. O'Leary (Eds.), *Encyclopedia of World Cultures* (Vol. 2: Oceania, pp. 65-68). New York: Macmillan Reference USA.

Regan AJ, Griffin H-M (2005) *Bougainville before the conflict*. Pandanus Books.

Routledge, D. (1996). Bau. In D. Levinson & T. O'Leary (Eds.), *Encyclopedia of World Cultures* (Vol. 2: Oceania, pp. 22-24). New York: Macmillan Reference USA.

Ryan, T. (2002). Niueans. In *Encyclopedia of World Cultures* (Vol. Supplement, pp. 227-230). New York: Macmillan Reference USA.

Saussol, A. (1971) New Caledonia: colonization and reaction. In: Crocombe R (ed). *Land tenure in the Pacific, third edition* (pp. 240-260). Suva: University of the Pacific Press.

Scheffler, H. (1996). Choiseul Island. In D. Levinson & T. O'Leary (Eds.), *Encyclopedia of World Cultures* (Vol. 2: Oceania, pp. 37-40). New York: Macmillan Reference USA.

Thomas, N. (1991). Marquesas Islands. In D. Levinson & T. O'Leary (Eds.), *Encyclopedia of World Cultures* (Vol. 2: Oceania, pp. 188-191). New York: Macmillan Reference USA.

Urbanowicz, C. (1996). Tonga. In D. Levinson & T. O'Leary (Eds.), *Encyclopedia of World Cultures* (Vol. 2: Oceania, pp. 336-339). New York: Macmillan Reference USA.

White, G. M. (1991). *Identity through History: Living Stories in a Solomon Islands Society*. Cambridge: Cambridge University Press.

White, G.M. (1997). The Discourse of Chiefs: Notes on a Melanesian Society. In G.M. White & L. Lindstrom. (Eds.), *Chiefs Today: Traditional Pacific Leadership and the Postcolonial State* (pp 229-252). Stanford, CA: Stanford University Press.
