# Supplementary material for: Cultural and Environmental Predictors of Pre-European Deforestation on Pacific Islands
Source: PLoS One. 2016 May 27;11(5):e0156340. doi: 10.1371/journal.pone.0156340 (PMC4883741; doi:10.1371/journal.pone.0156340)
Supplement: S5 Table — (PDF) [file pone.0156340.s007.pdf]

S5 Table. Estimates of phylogenetic signal (lambda) for each of the ecological and cultural predictors, averaged across 100 replicates from the posterior distribution of language trees.

Ecological Variables	Log likelihood without Lambda	Log likelihood with lambda	Lambda	p value
Abs. Latitude	-261.841	-209.945	0.998	0.000
Age	-52.433	-46.658	0.706	0.000
Dust	-490.270	-414.329	1.000	0.000
Log(Area)	-120.525	-112.034	0.776	0.000
Log(Elevation)	-51.120	-46.372	0.676	0.001
Log(Isolation)	-74.410	-74.206	0.119	0.509
Log(Rainfall)	-14.187	-4.741	0.407	0.000
Makatea	38.328	42.399	0.584	0.002
Tephra = 2	6.104	62.962	1.000	0.000
Tephra = 3	-53.437	29.206	1.000	0.000

Cultural Variables	Log likelihood without Lambda	Log likelihood with lambda	Lambda	p value
Wet	-54.737	-42.479	0.871	0.000
Tree	-42.835	-0.319	1.000	0.000
Dry	-53.848	-43.248	0.835	0.000
Elite	-49.617	3.031	1.000	0.000
Individual	-45.505	-4.985	1.000	0.000
