# Supplementary material for: Cultural and Environmental Predictors of Pre-European Deforestation on Pacific Islands
Source: PLoS One. 2016 May 27;11(5):e0156340. doi: 10.1371/journal.pone.0156340 (PMC4883741; doi:10.1371/journal.pone.0156340)
Supplement: S6 Table — (PDF) [file pone.0156340.s008.pdf]

S6 Table. Ecological predictors of forest replacement including cultural ancestry (lambda) but not geographic proximity (phi).

Predictor	Relative variable importance	Akaike Weighted Beta estimate	95% confidence interval
**Tephra = 3**	1.000	-1.167	(-1.573, 0.207)
**Abs. Latitude**	0.999	-0.034	(-0.048, 0.007)
**Log(Area)**	0.975	-0.208	(-0.296, 0.045)
**Dust**	0.898	-0.001	(-0.002, 0)
**Tephra = 2**	0.877	-0.720	(-1.227, 0.259)
**Log(Isolation)**	0.772	0.104	(0.029, 0.038)
**Log(Elevation)**	0.305	0.094	(-0.118, 0.108)
**Makatea**	0.295	0.170	(-0.22, 0.199)
**Age**	0.189	-0.032	(-0.136, 0.053)
Log(Rainfall)	0.177	0.041	(-0.065, 0.054)
Dependency	-	Mean	p-value
Lambda	-	0.991	<0.001
Independent	-	0.009	-

Table shows relative variable importance, akaike weighted beta estimate and 95% confidence interval on the mean for PGLS-spatial analysis of the effects of cultural ancestry and putative ecological predictors on forest replacement (n=72). Previously identified significant predictors of deforestation are shown in bold. All values integrate over phylogenetic and sampling uncertainty across 100 replicates from our posterior distribution of language trees.
