# Supplementary material for: Cultural and Environmental Predictors of Pre-European Deforestation on Pacific Islands
Source: PLoS One. 2016 May 27;11(5):e0156340. doi: 10.1371/journal.pone.0156340 (PMC4883741; doi:10.1371/journal.pone.0156340)
Supplement: S7 Table — (PDF) [file pone.0156340.s009.pdf]

S7 Table. Model of forest replacement including only cultural ancestry (lambda) and geographic proximity (phi).

Dependency	Mean	p-value
Lambda	0	1
Phi	1	<0.001
Independent	0	-

Table shows mean and p-values for PGLS-spatial analysis of the effects of cultural ancestry(lambda) and geographic proximity (phi) on forest replacement (n=72). All values integrate over phylogenetic and sampling uncertainty across 100 replicates from our posterior distribution of language trees.
