# Supplementary material for: Cultural and Environmental Predictors of Pre-European Deforestation on Pacific Islands
Source: PLoS One. 2016 May 27;11(5):e0156340. doi: 10.1371/journal.pone.0156340 (PMC4883741; doi:10.1371/journal.pone.0156340)
Supplement: S8 Table — (PDF) [file pone.0156340.s010.pdf]

**S8 Table**. **Descriptions of ecological variables**[1]	
Variable	Description
Rainfall(log)	Rainfall at sea level (mm/year)
Elevation(log)	Elevation of highest point of the island (m)
Area(log)	Area of the island (km^2)
Isolation(log)	Distance to nearest high island greater than 25% of the target island
Absolute Latitude	Absolute value of the island's latitude. Obtained from Google Earth
Makatea	Percentage of surface area composed of makatea. Makatea is sharp, rough, uplifted reef, which is not conducive to supporting vegetation
Age	Age of island estimated from volcanic rock. Raw data coded as 1) <20,000yrs; 2) 20,000 – 1,000,000yrs; 3) >1,000,000yrs or X) no volvanic rock dated. Rolett and Diamond (2004) only report an effect of a dummy variable coding Age=3, hence we use a binary coding representing the presence or absence of an age score of 3.
Tephra(2) and Tephra(3)	Aerial tephra is a measure of volcanic ash fallout. This was approximated using the island's position relative to the Andesite line. Coded as 1) Low (>1,000km east of the Andesite line); 2) Moderate (<1,000km east of the line); and 3) High (west of the line). Following Rolett and Diamond (2004), Tephra2 and Tephra3 are binary variables in which a score of 1 represents the presence of an aerial tephra score of 2 and 3 respectively
Dust	Asian dust fallout in mg/m^2/year

[1]Data for the above variables were taken directly from Rolett and Diamond (2004), except for the following: 1) we found an error in one of the original latitude values (Alofi Island), which was corrected using Google Earth; 2) there was no rainfall data from Alofi, so we used the rainfall for neighboiuring Futuna; 3) the area for alofi and Futune was made consistent with Kirch (1981); and 4) we included West Coast NZ from Rolett and Diamon's original source files.
