# Supplementary material for: Cultural and Environmental Predictors of Pre-European Deforestation on Pacific Islands
Source: PLoS One. 2016 May 27;11(5):e0156340. doi: 10.1371/journal.pone.0156340 (PMC4883741; doi:10.1371/journal.pone.0156340)
Supplement: S9 Table — (PDF) [file pone.0156340.s011.pdf]

S9 Table. Table showing results predicting deforestation and forest replacement from separate models including the intercept plus each cultural predictor, and controlling for the effects of cultural ancestry and spatial proximity.

Deforestation					Forest replacement				
**Predictor**	**Beta**	**95% C.I.**	**n**	**r-sqaured**	**Predictor**	**Beta**	**95% C.I.**	**n**	**r-sqaured**
Arboriculture	-0.441	-1.03, 0.148	80	0.027	Arboriculture	0.768	0.515, 1.021	76	0.323
Dry Intens.	-0.032	-0.429, 0.366	80	0.000	Dry Intens.	0.043	-0.05, 0.135	76	0.011
Elite ownership	-0.079	-0.748, 0.589	80	0.001	Elite ownership	0.538	0.199, 0.876	76	0.116
Ind. Ownership	-0.189	-0.623, 0.245	80	0.009	Ind. Ownership	0.031	-0.122, 0.184	76	0.002
Wet intens.	0.461	0.121, 0.801	80	0.083	Wet intens.	0.017	-0.057, 0.092	76	0.002

Table gives beta, 95% confidence interval, n and r-squared value for each analysis. All values integrate over phylogenetic and sampling uncertainty across 100 replicates from our posterior distribution of language trees.
