# Supplementary material for: Cultural and Environmental Predictors of Pre-European Deforestation on Pacific Islands
Source: PLoS One. 2016 May 27;11(5):e0156340. doi: 10.1371/journal.pone.0156340 (PMC4883741; doi:10.1371/journal.pone.0156340)
Supplement: S10 Table — (PDF) [file pone.0156340.s012.pdf]

Supplementary Table S10 - Ecological and cultural predictors of forest replacement (excluding Arboriculture).

Predictor	RVI	Beta	95% C.I.
Elite Ownership	0.988	0.606	0.271, 0.941
Log(Area)	0.89	-0.043	-0.074, -0.012
Abs. Latitude	0.457	-0.033	-0.072, 0.007
Tephra = 2	0.26	-0.394	-1.066, 0.278
Log(Isolation)	0.254	0.015	-0.018, 0.048
Tephra = 3	0.246	-0.435	-1.249, 0.38
Wet intens.	0.226	0.029	-0.047, 0.105
Dry intens.	0.2	0.024	-0.071, 0.119
Ind. Ownership	0.185	-0.019	-0.166, 0.127

Dependency	Mean	p-value
Cultural (λ')	0	1
Spatial (ϕ)	1	<0.001
Independent (γ)	0	-

Table showing relative variable importance, Akaike weighted beta estimate and 95% confidence interval for PGLS analysis of the effects of cultural and ecological predictors, phylogeny and spatial proximity on forest replacement (n=76). All values integrate over phylogenetic and sampling uncertainty across 100 replicates from our posterior distribution of language trees.
