# Supplementary material for: Cultural and Environmental Predictors of Pre-European Deforestation on Pacific Islands
Source: PLoS One. 2016 May 27;11(5):e0156340. doi: 10.1371/journal.pone.0156340 (PMC4883741; doi:10.1371/journal.pone.0156340)
Supplement: S11 Table — (PDF) [file pone.0156340.s013.pdf]

**S11 Table. Coding schemes for forest outcomes data based on descriptions from early European visitors.**		
Score	Deforestation	Replacement
1	No deforestation	Introduced species comprised less than 10% of all tree individuals
2	Densely forested	Introduced species comprised 25-50% of forest tree individuals up to 600m and less than 10% above 600m
3	Densely forested but with mainly introduced species in lowland in lowland secondary forests; much fire-associated grassland/ fernland on ridges, slopes and plateaus	Introduced tree species comprised 50-75% of forest tree individuals up to 600m and less than 10% above 600m, with less land above 600m than islands with a score of 2
4	Largely deforested; forests mainly on coastal plain, valley floors, and very steep slopes	As 3 but introduced tree species comprised 75-100% of forest tree individuals up to 600m
5	Almost completely deforested; fire-associated grassland/ fernland covering almost all areas not used as cropland	Original description: "There were few trees, and forest was mostly replaced by grasses and shrubs." We excluded the two sites with a score of 5 (Easter Island and Niihau Island) because it is not clear that a lack of any forest constitutes high levels of forest replacement.
