# Supplementary material for: Cultural and Environmental Predictors of Pre-European Deforestation on Pacific Islands
Source: PLoS One. 2016 May 27;11(5):e0156340. doi: 10.1371/journal.pone.0156340 (PMC4883741; doi:10.1371/journal.pone.0156340)
Supplement: S12 Table — (PDF) [file pone.0156340.s014.pdf]

S12 Table. Priors on divergence time of dialects and major clades imposed on Austronesian language phylogeny[1].

Clade	Constraint
Root age	3300-3800 BP (mean=3500, s.d.=100) based on previous estimates of age of clade from dated Austronesian language phylogeny (Gray, Drummond, & Greenhill, 2009)
Proto-Central Pacific	2900-3200 BP. Based on estimates for the earliest settlements of the Fiji/Tonga/Samoa region by approximately 2950 BP (Burley, Weisler, & Zhao, 2012).
Nuclear Polynesian	1400-2900 BP corresponding to the 'second long pause' in (Gray et al., 2009) in which Proto-Central Pacifc diverged into Proto Nuclear Polynesian.
Eastern Polynesian	1000-1400 BP (mean 1200, s.d.=100) based on radio carbon dates for the colonization of Eastern Polynesia (Wilmshurst, L., Lipo, & Anderson, 2011)
Rarotongan dialects (1-5)	180-620 BP. Mutually intelligible dialects, assumes a similar level of divergence to North vs. South Island Maori
Hawaiian dialects (1-14)	180-620 BP. Mutually intelligible dialects, assumes a similar level of divergence to North vs. South Island Maori
Futuna East dialects (1 & 2)	50-150 BP. Separated by only a 2km channel. No evidence of dialect variation. The age of the split between these two dialect variants should be less than that between identifiable dialects, such as Rurutuan/Tahitian.
Mangareva dialects (1 & 2)	50-150 BP. Same island. No evidence of dialect variation. The age of the split between these two dialect variants should be less than that between identifiable dialects, such as Rurutuan/Tahitian.
Samoan dialects (1 & 2)	50-150 BP. No significant dialect variation. The age of the split between these two dialect variants should be less than that between identifiable dialects, such as Rurutuan/Tahitian.

Fijian Bau dialects (1-6)	The age of this dialect group should be less than that age of its split with West Fijian Navosa
Marquesan Nukuhiva & Marquesan dialects (1, 5 & 6)	Allowed to branch off in any order within Northern Marquesan group
Marquesan dialects (2-4)	Allowed to branch off in any order within Southern Marquesan group
Rurutuan dialects (1-4)	Diversification must post-date split with Tahitian at 250 years
TahitianModern (1-7)	Diversification must post-date split with Rurutuan at 250 years

[1] Major clades are calibrated according to archaeological evidence. All of the dialect groups were assumed to be monophyletic. In addition, where more than one location was sampled on an island, the dialects from that island were treated as a subgroup. Marquesan was also separated into Northern and Southern Marquesan subgroups. Whilst some of the island groups involved have been settled for some time, we did not use the time of first settlement as athe age of the dialect group because long-term political and trade links have suppressed dialect variation. Dialect divergence dates are approximations based on evidence of relative divergence times compared to other dialects in our sample, together with our prior distribution on branch lengths. Fijian, Marquesan, Rurutuan and Tahitian dialect groups did not require age calibrations because close relatives of each group provided a natural constraint on their ages.

References

Burley, D., Weisler, M. I., & Zhao, J.-x. (2012). High Precision U/Th Dating of First Polynesian Settlement. *PLoS ONE, 7*(11), e48769. 10.1371/journal.pone.0048769

Gray, R. D., Drummond, A. J., & Greenhill, S. J. (2009). Language Phylogenies Reveal Expansion Pulses and Pauses in Pacific Settlement. *Science, 323*(5913), 479-483.

Wilmshurst, J. M., L., H. T., Lipo, C. P., & Anderson, A. J. (2011). High-precision radiocarbon dating shows recent and rapid initial human colonization of East Polynesia. *Proceedings of the National Academy of Sciences, 11*(5), 1815-1820. 10.1073/pnas.1015876108
