# Supplementary material for: Cultural and Environmental Predictors of Pre-European Deforestation on Pacific Islands
Source: PLoS One. 2016 May 27;11(5):e0156340. doi: 10.1371/journal.pone.0156340 (PMC4883741; doi:10.1371/journal.pone.0156340)
Supplement: S13 Table — (PDF) [file pone.0156340.s015.pdf]

S13 Table. Table showing results predicting deforestation and forest replacement from separate models including the intercept plus each ecological predictor, and controlling for the effects of cultural ancestry and spatial proximity.

Deforestation					Forest replacement				
**Predictor**	**Beta**	**95% C.I.**	**n**	**r-sqaured**	**Predictor**	**Beta**	**95% C.I.**	**n**	**r-sqaured**
Abs. Latitude	0.042	-0.009, 0.092	80	0.033	Abs. Latitude	-0.03	-0.072, 0.014	76	0.023
Age	0.498	0.153, 0.844	76	0.097	Age	-0.016	-0.094,0.063	72	0.002
Dust	0.002	-0.001, 0.006	80	0	Dust	-0.001	-0.003, 0.002	76	0.004
Log(Area)	-0.099	-0.176, -0.022	80	0.075	Log(Area)	-0.034	-0.067, -0.002	76	0.054
Log(Elevation)	-0.152	-0.311, 0.007	80	0.043	Log(Elevation)	-0.02	-0.084, 0.044	76	0.005
Log(Isolation)	0.042	-0.067, 0.15	80	0.007	Log(Isolation)	-0.004	-0.038, 0.03	76	0.001
Log(Rainfall)	-0.701	-0.914, -0.488	80	0.348	Log(Rainfall)	0.007	-0.038, 0.052	76	0.001
Tephra = 2	-0.746	-1.567, 0.075	80	0.039	Tephra = 2	-0.099	-0.608, 0.41	76	0.002
Tephra = 3	0.081	-0.284, 0.447	80	0	Tephra = 3	-0.13	-0.734, 0.476	76	0.002
% Makatea	-0.174	-1.119, 0.77	80	0.002	% Makatea	-0.624	-0.955, -0.292	76	0.155

Table gives beta, 95% confidence interval, n and r-squared value for each analysis. All values integrate over phylogenetic and sampling uncertainty across 100 replicates from our posterior distribution of language trees.
